# Exploring peptide dendrimers for intestinal lymphatic targeting: formulation and evaluation of peptide dendrimer conjugated liposomes for enhancing the oral bioavailability of Asenapine maleate

**DOI:** 10.1038/s41598-024-79372-5

**Published:** 2024-11-15

**Authors:** Ajjappla Basavaraj Shreya, Abhijeet Pandey, Sanjay Kulkarni, K Vijaya Bhaskar, Harendra S. Parekh, Srinivas Mutalik

**Affiliations:** 1 Department of Pharmaceutics, Manipal College of Pharmaceutical Sciences, Manipal Academy of Higher Education, Manipal, Karnataka 576104 India; 2 Global Drug Development/Technical Research and Development, Novartis Healthcare Pvt. Ltd., Genome Valley, Hyderabad, Telangana 500101 India; 3 Department of Pharmaceutical Chemistry, Manipal College of Pharmaceutical Sciences, Manipal Academy of Higher Education, Manipal, Karnataka 576104 India; 4 School of Pharmacy, Pharmacy Australia Centre of Excellence, The University of Queensland, Brisbane, QLD 4072 Australia

**Keywords:** Peptide dendrimer, Liposome, Lymphatic targeting, Schizophrenia, Neurological disorders, Drug delivery, Pharmaceutics, Pharmacology

## Abstract

Asenapine maleate (ASPM) is a second-generation atypical antipsychotic that is approved for treating acute schizophrenia and bipolar disorder in adults by the US FDA. The major downside of ASPM therapy is rapid, extensive first-pass hepatic metabolism following its oral administration with a very low oral bioavailability of < 2%. In this work, we developed ASPM nanoformulations conjugated with ligands such as arginine-glycine-aspartic acid (RGD) and peptide dendrimers (PDs) with the intention of improving the oral bioavailability of the drug by targeting it to the intestinal lymphatic system (ILS). Peptide dendrimers (PDs), both lipidated and nonlipidated, were synthesized by Fmoc solid phase peptide synthesis (SPPS). Reverse phase high performance chromatography (RP-HPLC) was used to purify the synthesized PDs, and the PDs were characterized by differential scanning calorimetry (DSC) electrospray ionization mass spectroscopy (ESI^+^-MS), Nuclear magnetic resonance (NMR) and Fourier transform infrared (FTIR) spectroscopy. The thin film hydration method was used to prepare liposomes, and the process variables affecting the liposome parameters were optimized using the Box‒Behnken design (BBD).Liposomes were PEGylated using DSPE-PEG-COOH_2000_ and further conjugated with ligands (RGD, PD-1 and PD-2) using EDC-NHS chemistry. The formulation was characterized using different spectroscopic techniques. In vitro, cell line studies, such as cytotoxicity, cell uptake, uptake mechanism, and receptor saturation studies, were performed on both Caco2 and Raji-B cells. The pharmacokinetic parameters of the developed liposomal formulation were evaluated using pharmacokinetic studies on Sprague- Dawley (SD) rats. The psychostimulant-induced hyperactivity model was used to evaluate the pharmacodynamic performance of the developed formulations by measuring the reversal of hyperlocomotor activity induced by levodopa-carbidopa.

**Supplementary Information:**

The online version contains supplementary material available at 10.1038/s41598-024-79372-5.

## Introduction

Schizophrenia is a chronic, heterogeneous, multifaceted cognitive and behavioral disorder characterized by a range of symptoms, including hallucinations, abnormal social behavior, delusions, impaired cognitive ability and disorganized speech/thought, which originate from the disruption of brain development due to environmental/genetic factors or both^1^. Bipolar disorder (BD) is a part of spectrum psychosis, a mood disorder that includes maniac episodes, hypomanic and major depressive episodes^2^. Recurrent episodes of depression and elevated mood accompanied by behavioural, cognitive and physical symptoms resulting in changes in energy level are the hallmark characteristics of BD^3^.

Antipsychotics are the most prescribed line of therapy for treating schizophrenia and BD, offering a more favourable tolerability profile and broader symptom control^4^. ASPM (Saphris®, USA; Sycrest®, UK) is a second (Atypical) generation antipsychotic belonging to the class of dibenzo-oxepino pyrrole that acts as a potent antagonist of dopamine, serotonin, noradrenaline, and histamine receptors. ASPM was approved for the treatment of acute schizophrenia and acute maniac or mixed episode associated with bipolar disorder-I in adults by the US FDA in August 2009^5^. The major drawback in ASPM therapy is that it undergoes rapid hepatic first metabolism upon oral administration, contributing to its low oral bioavailability (< 2%)^6^. Although the marketed fast-dissolving sublingual tablet of ASPM circumvents hepatic first-pass metabolism, its bioavailability is limited to only 35%. Patient noncompliance, anxiety, dysgeusia, akathisia, dyskinesia, oral hypoesthesia, and dizziness are some of the adverse effects associated with sublingual ASPM^7^. These drawbacks associated with the current ASPM therapy can be overcome by developing a formulation that can be administered via the oral route and by employing nanotechnology.

The oral route is widely regarded as the most natural, convenient and safest route of drug administration. The oral route is particularly advantageous for antipsychotic drugs, as these medications are often prescribed for a prolonged period of time, thus increasing patient adherence to therapy. In recent years, drugs targeting the intestinal lymphatic system (ILS) have emerged as promising alternative pathways for oral drug delivery. By targeting the ILS, drugs can bypass hepatic first-pass metabolism and can be absorbed directly into the systemic circulation, which is especially beneficial for drugs with poor oral bioavailability^8^. Nanocarriers offer a range of advantages for improving oral drug delivery and targeting drug moieties to the ILS, including enhanced surface area, size control, biocompatibility, enhanced permeation, and site-specific targeting through surface modification. Additionally, nanocarriers also enhance the biodistribution of drugs, which plays a crucial role in increasing the therapeutic efficacy of drugs^9^.

Among nanocarriers, liposomes stand out as highly effective vehicles for ILS targeting and are widely explored. Liposomes are self-assembled, spherical soft matter vesicles that are characterized by multiple concentric bilayers of lipids with an aqueous phase inside and between lipid bilayers^10^. Liposomes can access the ILS through the chylomicron-assisted pathway via lacteals or via the M-cell-mediated pathway through Peyer’s patches. They also enhance drug uptake via the ILS by absorbing at the gut-associated lymphoid tissue (GALT) containing Peyer’s patches and isolated lymphoid follicles^11–13^.

The Peyer’s patches (PPs) present in the intestinal epithelial layer serve as a door for the targeting and uptake of drugs via the ILS. The follicle-associated epithelium (FAE) present in the B cells of PPs encompasses targeting sites such as M cells, enterocytes and goblet cells. Active targeting strategies using specific ligands can be used to target M cells^12^. Specific targeting and enhanced uptake of drugs with reduced toxicity can be achieved by using M-cell-mediated targeting of nanocarriers to PPs conjugated with specific ligands. Various ligands are utilized to target M cells and thereby increase cellular uptake and drug bioavailability. Arginine-glycine-aspartic acid (RGD) is a ligand that is widely used for targeting FAEs overlying Peyer’s patches.

RGD is a peptide with a linear sequence that is used to target overexpressed β_1_ integrins on the apical side of M-cells in PPs of the ILS. Since the RGD peptide can bind to multiple integrin species, it is widely used as a targeting agent for delivering bioactive agents and drugs. Peptide dendrimers (PDs) are macromolecules with wedge or radial-like structures composed of peptides as their core branching unit and as covalently bonded terminal functional groups^14^. The term “peptide dendrimer” is derived from the structural attributes of these molecules that combines the elements of both peptides and dendrimer. The term “peptide” refers to the presence of peptide bonds formed between the amino acids, while the term “dendrimer” refers to their tree like branched architecture^15^. The globular shape of the PDs, which offers the desired dendritic topology is obtained through synthesis using solid phase peptide synthesis (SPPS) method^16^. They are widely explored as drug carriers and ligands because of their unique properties, such as solubility, chemical stability, multivalency and electrostatic interactions^17^. The main reason for selecting PDs as conjugating ligands is because they are biodegradable and biocompatible because they are composed of amino acids, which are considered as the cell building blocks. Furthermore, the presence of amino acid groups such as arginine and lysine will help reduce the cytotoxicity and cellular uptake of PDs^18^, and we also wanted to explore the use of PDs as ligands to target the M-cells of Peyer’s patches.

In the past, many researchers have endeavoured to improve the oral bioavailability of ASPM by formulating biodegradable long-acting injectable ASPM implants using PLGA for uniform drug release for 21 days^19^, a nanotransferosomal gel with a chemical permeation enhancer to increase the bioavailability of ASPM via the transdermal route^20^, a nanostructured lipid carrier to increase the oral bioavailability of ASPM by targeting to ILS^21^, RGD peptide-conjugated ASPM liposomes targeted to PPs for improving the lymphatic delivery of ASPM^22^, peptide dendrimer-conjugated liposomes to improve the transdermal delivery of ASPM^23^, solid lipid nanoparticles to improve the oral bioavailability of ASPM^24^, PLGA microspheres prepared by a microfluidic technique for the sustained release of ASPM^25^ and invasomes containing terpenes as natural permeation enhancers for the transdermal delivery of ASPM to improve its bioavailability and therapeutic efficacy^26^. Apart from these nanoformulations, there are scarcely any reports available on the use of PDs for targeting ILS and for the efficient oral delivery of ASPM.

The novelty of the present study lies in exploring peptide dendrimers for integrin-targeted delivery of lipid-based nanovesicles for enhanced internalization and bioavailability of ASPM. This study aimed to develop PD-conjugated (lipidated and nonlipidated) ASPM liposomes for M cell receptor-mediated targeting of ILS to enhance oral bioavailability. This study also attempted to elucidate the exact mechanism of uptake of PD-ASPM-conjugated liposomes via different pathways and to compare the efficacy of both RGD-conjugated liposomes and PD-conjugated liposomes in targeting ILS. Overall, these novel ligand-targeted ASPM liposomes will serve as a more effective treatment modality by enhancing the oral bioavailability of ASPM for treating schizophrenia and bipolar disorder.

## Materials and methods

### Materials

ASPM was obtained as a generous gift sample from MSN Organic Pvt. Ltd. (Hyderabad, India) and Orbicular Pharm Tech R&D (Hyderabad, India). Lipids such as cholesterol (CHO) were purchased from Merck Pvt. Ltd (Mumbai, India) 1,2-Dipalmitoyl-sn-glyceo-3-phosphocholine (DPPC) was obtained from Avanti Polar Lipids, Inc. (Alabama, USA), 1,2-distearoyl-sn-glycero-3-phosphoethanolamine-N-[carboxy (polyethylene glycol)-2000] (ammonium salt) (DSPE-PEG_2000_-COOH) was obtained from Nanocs, Inc. (New York, USA), and soya phosphatidyl choline (SPC) was purchased from Sigma Aldrich (St. Louis, MO, USA). Arginine-glycine-aspartic acid (RGD) was purchased from Sigma Aldrich (St. Louis, MO, USA). Dulbecco’s modified Eagle’s medium–high glucose media (DMEM) was procured from Gibco-Thermo Fischer Scientific Inc. (Mumbai, India), and Dulbecco’s phosphate-buffered saline (D-PBS), fetal bovine serum (FBS) and Roswell Park Memorial Institute Medium-1640 (RPMI-1640) were purchased from Himedia Laboratories Pvt, Ltd. (Mumbai, India). Fluorescein 5-isothiocyanate (FITC) and MTT (3-(4,5-dimethylthiazol-2-yl)-2,5-diphenyltetrazolium bromide) were procured from Sigma Aldrich (St. Louis, MO, USA). The human epithelial colorectal adenocarcinoma (Caco2) and human Burkitt’s lymphoma B lymphocyte (Raji-B) cell lines used in the cell line studies were procured from the National Centre for Cell Science (Pune, India). N-(3-Dimethylaminopropyl)-N′-ethyl carbodiimide hydrochloride (EDC. HCl) and N-hydroxysuccinimide (NHS) were obtained from Tokyo Chemical Industries (Tokyo, Japan), and 2-(N-morpholino) ethanesulfonic acid buffer (MES) was obtained from Sigma Aldrich (St. Louis, MO, USA). Levodopa/Carbidopa 100/25 mg tablets (Syndopa Plus; Sun Pharmaceutical Industries Ltd., Mumbai, India) were purchased from Pharmacy. All the other solvents, chemicals and reagents used in the formulation were of analytical or HPLC grade.

### Synthesis, purification and characterization of Peptide Dendrimers.

The synthesis of arginine-terminated, lipidated PD (lipidated with a decanoic acid (C_10_) lipid chain) with a positive charge (R4: 4+) and a C-N sequence of glycine-leucine-lysine (decanoic acid)-lysine-(arginine)_2_ (Gly-Leu-Lys(C_10_)-Lys-(Arg)_2_; termed PD-1) and nonlipidated, arginine-terminated PD with a positive charge (R4: 4+) and a C-to-N sequence of glycine-leucine-lysine-(arginine)_2_ (Gly-Leu- Lys-(Arg)_2_; termed PD-2) was carried out using Fmoc-Solid phase peptide synthesis (SPSS) according to methods reported previously^23,27^. Briefly, chlorotrityl chloride resin was used as a solid support, and preactivated (protected) Fmoc-amino acids were sequentially coupled to this solid support. The coupling efficiency of amino acids was determined by the ninhydrin test at each amino acid coupling step as this test is highly sensitive, simple and reliable^28^. The next amino acid was coupled only after achieving 99% of the coupling efficacy of the previous amino acid^29^. This series of deprotection and coupling of amino acids was performed continuously until the desired chain length of the PD was obtained. Lipidated PDs were synthesized by coupling a lipid preactivated decanoic acid (C_10_) chain to amino acids followed by sequential building of the amino acid onto a solid support^30^. The PDs of the desired chain length were cleaved from the resin solid support under acidic conditions, followed by filtration, and the resulting PD was lyophilized and stored at 2–8 °C until further use.

The synthesized PDs were purified by using preparative RP-HPLC (Shimadzu Corporation, Kyoto, Japan). Lipidated PD (PD-1) was purified using a Sepax GP C-18 (5 µm, 120 Å, 4.6 * 250 mm) column, whereas for the purification of nonlipidated PD (PD-2), Kromasil C_18_ (5 µm, 4.6 * 250 mm) was used. Characterization of the synthesized PDs was performed by electrospray ionization mass spectrometry (EDI^+^ MS) (Shimadzu EVLCMS-2010, Shimadzu Corporation, Kyoto, Japan) to determine the molecular weight of the PDs. The structural dynamics of the PDs were determined by NMR (AV-400, Bruker, USA). The PDs were further characterized by Fourier transform infrared spectroscopy (FT-IR) (FTIR-8300, Shimadzu Corporation, Kyoto, Japan) to identify the functional groups present in the PDs and by differential scanning calorimetry (DSC) (DSC-60, Shimadzu Corporation, Kyoto, Japan) to determine the melting points of the amino acids present in the PDs.

### Preparation of ASPM-loaded liposomes

Drug- Excipient compatibility studies were performed to assess the compatibility between ASPM and the excipients used in the formulation of liposomes using FT-IR. The results of compatibility studies are discussed in Supplementary information; Section S4; Figure S8. ASPM-loaded liposomes (LP-ASPM) were prepared using a previously reported thin film hydration technique^31^, with slight modifications to the process variables. Briefly, ASPM and cholesterol (CHO) were dissolved in a mixture of chloroform and methanol (9:1 v/v) in a round bottom flask (RBF). Lipids such as SPC and DPPC were completely dissolved by manual shaking. The lipid solution was vacuum evaporated (R-215, Buchi, Switzerland) at 45 °C and at a rotation speed of 80 RPM for 30 min to form a thin film. The thin lipid film was dried in a vacuum desiccator and then hydrated by adding 10 mL of pH 7.4 phosphate buffer. The obtained milky white liposomal dispersion was further probe sonicated (Vibra Cell™, VC-130, Sonics and Material, Inc., USA) in an ice bath to obtain vesicles of uniform particle size. The unencapsulated drug was removed by centrifuging the liposomal dispersion at 22,000 rpm for 1 h at 4 °C. The obtained liposomal formulation was stored at 4 °C until further use^22,32^. Separating liposomes from unencapsulated drugs and other components is sufficiently effective without causing significant damage to the liposomes at 22,000 rpm. Additionally, at this speed, the liposomes can be easily pelleted, as the buoyant force exerted by the surrounding medium on the liposomes will be high, allowing the liposomes to settle down^33^.

### Optimization and validation of ASPM liposomal formulation

The one-factor-at-a-time (OFAT) method was used to identify these critical factors (Supplementary information; Section S5; Table S5, S6, S7, S8, S9 and S10). The other process parameters, such as the hydration media and volume (pH 7.4 phosphate buffer/10 ml), rotary evaporator parameters (45 °C/80 rpm) and amount of drug (5 mg) added, were kept constant. The effect of the selected critical factors (independent variables) and their interactions on the liposome properties was statistically optimized by a 3-factor, 3-level Box‒Behnken design (BBD) using Design Expert® software (v.10.0.3.1; Stat-Ease, Inc., Minneapolis, USA). Total lipid content-X_1_ (mg), sonication time-X_2_ (min) and Pulser “on time”-X_3_ (sec) were the independent variables evaluated (Supplementary information; Section S6a; Table S11). A design matrix comprising 15 experimental trials with 3 replicates at the centre point was constructed to optimize the 3 selected independent variables (Supplementary information; Section S6a; Table S12). The liposomal attributes (dependent variables), such as the particle size -Y_1_ and entrapment efficiency (%EE) of ASPM-Y_2_ were selected as the response variables. Analysis of variance (ANOVA) using Fisher’s F test was carried out to determine the statistical significance of the model. The appropriateness of the model was statistically verified by using the coefficient of determination (R^2^) and adjusted R^2^, while Minitab® software (v 19.2.0, Minitab LLC, Pennsylvania, USA) was used to assess the desirability and goodness of fit of the model.

#### Effect of the amount of drug on the optimized ASPM liposomes

The optimization was achieved by increasing the amount of drug in the liposome formulation. Liposomes containing 10 mg, 15 mg, 20 mg, or 25 mg of drug were prepared under optimized conditions to study the effect of different amounts of drug on particle size, poly dispersity index (PDI), zeta potential (ZP) and %EE. The optimal amount of drug to be loaded in the liposomal formulation was selected based on the particle size, PDI, ZP, %EE and loading efficiency (%LE).

#### Effect of different cryoprotectants on optimized ASPM liposomes

The pellets of the optimized LP‒ASPM dispersion were redispersed using Milli-Q water. Five percent (w/w) cryoprotectants, viz*.,* mannitol, trehalose and sucrose, were dissolved in 500 µL of Milli-Q water and added to the redispersed liposomes. The liposomes containing cryoprotectants were frozen at − 80 °C for 12 h, followed by freeze drying (Martin Crist, Germany). The lyophilized liposomes were then reconstituted with Milli-Q water, and the PDI, particle size and ZP were measured. The optimum cryoprotectant to be used in the formulation of liposomes was selected based on the change in particle size of the liposomes before and after lyophilization.

### Surface modification of LP-ASPM by EDC-NHS chemistry

The surface modifications of the liposomes were carried out by carbodiimide chemistry. The targeting ligands (RGD/PDs) were conjugated to the surface of LP-ASPM using EDC-NHS via covalent conjugation, and DSPE-PEG-COOH_2000_ was used as a linker. PEGylated liposomes (LP-DSPE-PEG) were prepared by replacing a specified concentration of DPPC from the optimized formula with that of DSPE-PEG-COOH_2000_. A suitable concentration of DSPE-PEG-COOH_2000_ for conjugation was selected by formulating liposomes with different concentrations of DSPE-PEG-COOH_2000_ (2%, 5%, 8% and 10%), and based on the particle size, ZP and PDI, the concentration of DSPE-PEG-COOH_2000_ required for conjugation was selected^34^.

The free carboxyl moieties present within the PEG ends of the PEGylated liposomes were conjugated with the amino groups present on the RGD and PDs. Briefly, EDC (2.2 mg) dissolved in MES buffer (0.1 M, pH 5) was added to the optimized PEGylated liposomes, which were stirred for 30 min. After incubation with EDC, NHS (1.11 mg, 2:1 EDC: NHS ratio) dissolved in MES buffer (0.1 M, pH 5) was added. The PEGylated liposomal dispersion containing EDC-NHS was continuously stirred at RT for 12 h to facilitate the formation of an amine reactive intermediate. The unreacted EDC, NHS was removed by dialysis against Milli-Q water using pre wetted dialysis membrane of 12,000 Da molecular weight cut off (MWCO), kept under stirring for 6 h. To this liposomal dispersion with an amine reactive terminal, RGD/PD-1 or PD-2 (5 times more than DSPE-PEG-COOH_2000_) dissolved in MES buffer (0.1 M, pH 5) was added, and the mixture was gently rotated for 24 h for conjugation. The excess unreacted reactants, RGD and PDs were removed by dialysis. A similar procedure was followed to prepare FITC-labelled conjugated liposomes to study the cellular uptake of ASPM^35,36^.

### Characterization of unconjugated and conjugated ASPM liposomes

The average particle size, PDI and zeta potential of the liposomes were determined by dynamic light scattering (DLS) using a Malvern Zeta sizer (Nano ZS ZEN 3600, Malvern Instruments, UK). Fourier transform infrared spectroscopy (FTIR) was performed using an FTIR spectrophotometer (FTIR-8300, Shimadzu Corporation, Kyoto, Japan) to confirm the conjugation of the ligands to the ASPM-loaded liposomes. Proton (^1^H) NMR studies were performed to further confirm the conjugation of RGD, PD-1 and PD-2 to the ASPM liposomes. Appropriate amounts of DSPE-PEG-COOH_2000_ and lyophilized LP-RGD, LP-PD-1 and LP-PD-2 were weighed and dissolved in dimethyl sulfoxide, and the analysis was carried out using a 400 MHz NMR instrument (AV-400, Bruker, USA). The thermal analysis of pure ASPM, plain liposomes, LP-ASPM and conjugated liposomes was performed by using DSC (DSC-60, Shimadzu Corporation, Kyoto, Japan). DSC thermograms of the samples were obtained by measuring heat flow as a function of temperature. The XRD patterns of the pure drug (ASPM), LP-ASPM, LP-DSPE-PEG, LP-RGD, LP-PD-1 and LP-PD-2 were obtained using an X-ray diffractometer (Ultima IV, Rigaku Corporation, Tokyo, Japan). The analysis was performed in continuous scan mode with a scanning speed of 3°/min at a scanning axis of 2Ɵ angles of 5°–85°.

The shape and surface morphology of the LP-ASPM, LP-DSPE-PEG, LP-RGD, LP-PD-1 and LP-PD-2 liposomes were studied using TEM (CM200 Supertwin system, Philip, Amsterdam, Netherlands). Briefly, a drop of the sample was placed on a copper grid coated with carbon film and air-dried for 1 min. The sample was then stained with 1% w/v phosphotungstic solution (pH 6.0) followed by drying under an IR lamp for 30 min. The sample-loaded copper grid was then analysed at a voltage of 200 kV and a resolution of 0.23 nm. The size of the liposomes, concentration and number of particles present in the liposome dispersion were determined by using a Malvern NanoSight instrument (NanoSight, NTA 3.4, Malvern Instruments, UK)^37^.

### Drug loading studies

The amount of ASPM encapsulated in liposomes was determined by the RP-HPLC method developed for the estimation of ASPM (*Supplementary information; Section S2*). Any amount of unentrapped ASPM in the liposomal dispersion was separated by centrifugation for 1 h at 22,000 rpm and 4 ºC. The collected supernatant was again centrifuged under the same conditions to remove any traces of unentrapped ASPM. The pellets obtained by the two centrifugation cycles were ruptured using Triton X-100 solution (1% w/v). The pellet dispersion was vortexed for 1 min for lipolysis to occur. To this mixture, methanol was added, and the sample was sonicated using a bath sonicator for 30 min to extract ASPM. The solution was then centrifuged for 10 min at 10,000 rpm, and the resulting supernatant was diluted with mobile phase buffer and filtered through a 0.22 µm membrane filter before injection into the HPLC system^38^. The encapsulation efficiency was calculated in accordance with the following equation:

$$\begin{aligned}&\text{Encapsulation efficiency }\left({\%}\right) =\frac{\text{Amount of ASPM enapsulated }}{\text{Total amount of ASPM added} } \times 100\end{aligned}$$

The amount of ASPM as a percentage of the total amount of liposomes is called the loading efficiency. To determine %LE, the liposomes were treated in the same way as mentioned in the procedure for calculating %EE and were injected into the HPLC system. The %LE of ASPM was calculated using the following formula^39^:

$$\begin{aligned}\%LE&=\frac{\text{Amount of ASPM in liposomes}}{\text{Total amount of liposomes}} &\times 100\end{aligned}$$

### In vitro drug release studies

The in vitro release pattern of ASPM from liposomes was studied using a dialysis method. The release studies were performed in pH 1.2 hydrochloric acid (HCl) solution for 2 h followed by studies in pH 4.5 ammonium acetate buffer for 1 h, pH 6.8 phosphate buffer for 6 h and pH 7.4 phosphate buffer for up to 48 h. The liposomal dispersion (equivalent to 2 mg of drug) was filled in pre-soaked dialysis bags (12,000 Da MWCO). The bags were suspended in a beaker containing 100 mL of each medium, and the mixture was placed on a magnetic stirrer for continuous stirring at 100 rpm at 37 °C ± 0.5 °C. An aliquot of 2 mL was collected and replaced with the same volume of fresh media. The release of the drug from the withdrawn samples was analysed using HPLC ^22^. The details of HPLC analytical method development and validation to estimate the amount of ASPM in liposomes are given in Supplementary information (Section S2; Table S1, S2, S3; Figure S4, S5 and S6).

### Maintenance of cell culture

Raji-B (human Burkitt’s lymphoma cell line) and Caco-2 (human colorectal adenocarcinoma cell line) cells were used for in vitro cell line studies. The cells were maintained in Roswell Park Memorial Institute (RPMI-1640) media and Dulbecco’s modified Eagle medium (DMEM) high glucose media supplemented with 10% FBS and 1% antibiotic–antimycotic solution under atmospheric conditions of 5% CO_2_ and 18–20% O_2_ at 37 °C in a CO_2_ incubator and sub cultured every 2 days.

#### In vitro cell viability assay

MTT assays were performed for liposomes (Plain and conjugated) in both Caco-2 and Raji B cells to assess the viability of the formulated liposomes. The assay was carried out once the cells reached confluency. Caco-2 and Raji B cells were seeded into the wells of 96-well plates at a concentration of 1 × 10^5^ cells/cm^2^ and incubated for 24 h at 37 °C and 5% CO_2_. The old medium was discarded at the end of 24 h, and the cells were incubated with various concentrations of liposomes, which were diluted with complete media to yield varying doses of 0.312–10 μg/mL for a period of 24 h at 37 °C and 5% CO_2_. Untreated cells in each experiment served as a cell control. After incubation, 20 μL of MTT solution (5 mg/mL) was added to each treated and untreated well, and the cells were incubated at 37 °C overnight in the dark. Here, formazan crystals are formed upon the reaction of MTT with the mitochondrial dehydrogenase enzyme present only in live cells. Then, 150 μL of DMSO was added to the cells to dissolve the purple-coloured MTT formazan crystals formed. The dissolution of the formazan crystals in DMSO was facilitated by oscillating the plates for 15 min, followed by measuring the absorbance immediately at 570 nm using a microplate reader (ELX-800, BioTek Instruments Inc., USA)^40^.

#### Cellular uptake studies

The uptake of the formulated liposomes was evaluated in both Raji B and Caco-2 cells. Upon reaching confluency, Caco-2 and Raji-B cells were seeded in a 6-well plate at a concentration of 2 × 10^5^ cells/cm^2^ and incubated for 24 h at 37 °C and 5% CO_2_. After 24 h, the cells were incubated with plain FITC (control) and various FITC-labelled liposomes (unconjugated, RGD, PD-1 and PD-2 conjugated) diluted with media (1 µg/mL) for a period of 2 h. The cells were detached using trypsin, and the cell suspension was centrifuged at 500×*g* for 5 min. The obtained pellets were suspended in culture media, and fluorescence was recorded using a FACS Calibur flow cytometer (BD FACS calibur) at an excitation wavelength of 488 nm^22,41^.

#### Mechanism of cellular uptake

The cellular internalization mechanism of the formulated liposomes was evaluated by an uptake poisoning protocol in both the Raji B and Caco-2 cell monolayers. The cells were seeded at a density of 2 × 10^5^ cells/mL in a 6-well plate and incubated for 48 h. The cells were initially treated with PBS (control), chlorpromazine (50 μM) for inhibiting clathrin-based endocytosis, amiloride (50 μM) for inhibiting micropinocytosis, nystatin (200 μM) for inhibiting caveolae-based endocytosis and sodium azide for inhibiting energy-dependent endocytosis for 1 h after incubation in DMEM for 30 min. A 10 μg/mL FITC-labelled liposomal formulation (unconjugated, RGD, PD-1 and PD-2 conjugated) was added to the treated cells and incubated for 2 h. Trypsin was used to detach the cells, and the cell suspension was centrifuged at 500xg for 5 min. The obtained pellets were suspended in culture media, and fluorescence was recorded using a FACS Calibur flow cytometer at an excitation wavelength of 488 nm^42^.

#### Receptor saturation study

The role of surface ligands in facilitating the uptake of nano formulations inside cells was assessed by performing a receptor saturation study on Raji B cells. The cells were seeded at a density of 2 × 10^5^ cells/mL in a 6-well plate and incubated for 48 h. The cells were initially treated with PBS (control) and preincubated with RGD and peptide dendrimers (PD-1 and PD-2) for 1 h after which they were incubated in DMEM for 30 min. Then, 10 μg/mL of the FITC-labelled liposomal formulation (unconjugated, RGD, PD-1 and PD-2 conjugated) was added, and the mixture was incubated for 2 h. Trypsin was used to detach the cells, and the cell suspension was centrifuged at 500 × g for 5 min. The resulting pellets were suspended in culture media, and fluorescence was recorded using a FACS Calibur flow cytometer at an excitation wavelength of 488 nm^43^.

#### Animal studies

Preclinical ex vivo and in vivo studies were conducted on male Sprague–Dawley (SD) rats (200–250 g). The SD rats were bred at the Central Animal Research Facility, Manipal Academy of Higher Education, Manipal, India. The SD rats were exposed to a light–dark cycle (12 h each) and had free access to food and water. The rats used in the study were housed in cages at 25 ± 2 °C. All the experimental procedures and animal handling were carried out following the CCSEA (Committee for Control and Supervision of Experiments on Animals, Ministry of Fisheries, Animal Husbandry and Dairying, Government of India) guidelines, and in accordance with ARRIVE guidelines as well. All the animal experimental protocols were approved by the Institutional Animal Ethical Committee, Kasturba Medical College, MAHE, Manipal (Approval No: IAEC/KMC/49/2020). An attempt was made to use the minimum number of rats in an ethically responsible manner.

#### Ex vivo drug permeation studies

The absorption of the drug through the ileum portion of the small intestine was determined by an ex vivo drug permeation study using an everted ileum sac model. Overnight-fasted SD rats were sacrificed humanely, and the small intestine was isolated (≈ 10 cm). The isolated ileum segment was carefully everted and pushed completely over a capillary tube. The ileum was then tied between the cannulated parts of the U-shaped glass tube, which was used as a perfusion apparatus. The perfusion apparatus was then placed in a beaker containing 450 mL of Ringer’s solution (pH 7.4). The medium was stirred using magnetic beads at 100 rpm and 37 °C. Plain ASPM solution/liposomal dispersion was added to the beaker. The samples were withdrawn from the cannulated arm of the U tube at predetermined time points of 1, 2 and 3 h, and the same volume of the withdrawn sample was replaced by the addition of fresh Ringer’s solution. The amount of drug absorbed was calculated by analysing the samples using HPLC^22^.

#### Pharmacokinetic studies

Overnight-fasted SD rats were divided into 3 groups (n = 6). Group I, ASPM solution; Group II, LP-ASPM; Group III, LP-PD-2. All the groups received ASPM at a dose of 5 mg/kg body weight orally. Blood was drawn from the retro-orbital sinus at different time points between 0 and 48 h in tubes containing disodium EDTA. Blood was centrifuged at 5000 rpm for 10 min, and the clear plasma from the supernatant layer was collected and stored at -80 °C until analysis. The amount of ASPM in the samples was analysed by RP-HPLC. The detailed bioanalytical method development for the quantification of ASPM in rat plasma is given in Supplementary information; Section S3; Table S4; Figure S7. Pharmacokinetic parameters were calculated using PK Solutions 2.0™ (Summit Research Services, Montrose, Colorado).

#### Pharmacodynamic studies

The pharmacodynamic study was performed using an induced locomotor activity (LMA) test with a psychostimulant-induced hyperactivity model. The ability of the optimized formulation to reverse hyperactivity in rats was studied. The SD rats were divided into the following treatment groups (n = 6): Group I: Normal group with no treatment; Group II: Positive control group administered L-DOPA (10 mg/kg) and carbidopa (2.5 mg/kg) intraperitoneally; Group III: L-DOPA-carbidopa-treated rats treated with ASPM solution (5 mg/kg; p.o.); Group IV: L-DOPA-carbidopa-treated rats treated with LP-ASPM (equivalent to ASPM dose 5 mg/kg; p.o.); Group V: L-DOPA-carbidopa-treated rats treated with LP-PD-2 (equivalent to ASPM dose: 5 mg/kg; p.o.). A digital Actophotometer (Ikon Instruments, New Delhi, India) was used to assess LMA at 5 min intervals at 1, 2 and 3 h. The LMA was assessed after 1 h of L-DOPA-carbidopa administration in group II. ASPM was administered after 30 min of L-DOPA-carbidopa administration, and LMA was noted after 30 min of ASPM administration in groups III, IV and V.

## Results

### Synthesis, purification and characterization of peptide dendrimers

Arginine (Arg)-terminated lipidated peptide dendrimers (PD-1; 4^+^ charged; lipidated with decanoic acid; C_10_) (PD-1) and Arg-terminated nonlipidated peptide dendrimers (PD-2; 4^+^ charged) were synthesized by Fmoc solid-phase peptide synthesis. The molecular weight of the synthesized PD-1 was 911.21 g/mol, with a sequence following Gly-Leu-Lys (C_10_)-Lys-(Arg)_2_ from the C-N-terminus. The sequence of synthesized PD-2 was Gly-Leu-Lys-(Arg)_2_ from the C-N-terminus, with a molecular weight of 629.30 g/mol. The percentage yield of the synthesized PDs was > 75%. A single peak was obtained by HPLC (Supplementary Information; Figure S1) for both PDs following purification and was collected within 15 min of the total run time, indicating a purity of > 95% for both PDs (Supplementary Information; Figure S1A,B). The ESI^+^-MS (Supplementary Information; Figure S1C,D) for both PDs showed the desired molecular ion ([M + H]^+^) peaks^44,45^.

The synthesized PDs were characterized by FTIR, DSC and NMR. The FTIR spectrum of PD-1 (Supplementary Information; Figure S2A) showed an intense sharp peak at 1626.17 cm^−1^ for the amide carbonyl group (-C = O-NH_2_) present between lysine and the decanoic acid (C_10_) side chain, indicating the conjugation between lysine and decanoic acid, and the intense sharp peak suggested that decanoic acid exists as a side chain. The peak observed at 1663.82 cm^−1^ was attributed to the carbonyl group (–C=O) of PD. Broad peaks for the aliphatic (-CH_2_) carbon chain (SP_3_ hybridization stretch) of decanoic acid were observed below 3000 cm^−1^, and a peak for the aliphatic chain (-CH_2_) of lysine was observed at 3265.95 cm^−1^^46^. The FTIR spectrum of PD-2 (Supplementary Information; Figure S2B) showed a peak at 1538.23 cm^−1^ (–OH) due to the bending vibration of the hydroxyl group of arginine. A peak at 1333.81 cm^−1^ was attributed to the symmetric plain bending of the methyl (–CH_3_) group present in arginine. A broad peak in the range of 2800–3200 cm^−1^ was observed due to the presence of small aliphatic (–CH_2_) chains of glycine and leucine. The DSC thermograms of PD-1 and PD-2 (Supplementary Information; Figure S2C,D) exhibited endothermic peaks in the range of 200–230 °C corresponding to the melting points of the amino acids glycine (233 °C), lysine (215 °C) and arginine (222 °C)^47^.

The ^13^C NMR spectrum of PD-1 (Supplementary Information; Figure S3A) showed a sharp intense signal at 12 ppm, representing the –CH_3_ group of the C_10_ side chain. The structure of PD-1 contains four types of ^13^C nuclei in the dendrimer, viz*.,* methyl (-CH_3_), methylene (–CH_2_), methine (-CH), and carbonyl (–C=O) groups and quaternary carbon groups. The signal peaks for the –CH_2_ groups of the C_10_ side chain were observed in the range of 20–30 ppm. The signal peak for the methyl group of the isopropyl group of leucine was observed at 22.5 ppm. The peaks observed in the signal range of 160–180 ppm represent the amide carbonyl groups of the amide linkages in PD-1. The –CH_2_ groups of lysine and glycine were observed at 118.78 ppm. The signal peak at 39 ppm refers to the carbon of -CH_2_ groups bonded to nitrogen atoms, and the signal peak at 40 ppm represents the solvent peak of DMSO.

The ^1^H-NMR spectrum of PD-2 (Supplementary Information; Figure S3B) showed a doublet peak at 0.8 ppm, representing the hydrogens of the isopropyl group of leucine. Similarly, a multiplet signal attributed to the -CH protons of the isopropyl group of lysine was observed at 1.5 ppm. The proton of the -NH group present in the amide bonds of the PDs was observed at a signal of 3.2 ppm. A signal peak for the –CH_2_ group protons of glycine was observed at 4.3 ppm. A triplet signal in the range of 3.5–3.8 ppm was observed for the -CH_2_ group protons of arginine. The –CH protons of arginine were observed at a signal of 1.8 ppm. The ^11^H-NMR signals for arginine residues were observed at 4 ppm, and the solvent peak of D_2_O was observed at 4.8 ppm.

### Formulation and characterization of liposomes

#### Optimization and validation of ASPM liposomal formulations

The thin film hydration method was employed to prepare liposomes, and the process variables affecting the liposome parameters were selected by the OFAT method (Supplementary information; Section S5). The effects of various process variables, such as the type of phospholipid, phospholipid ratio, total lipid content, sonication time, pulser “on time” and hydration media on liposome parameters such as particle size, ZP and PDI were studied. The process variables (total lipid content, sonication time and pulser “on time”) that had a positive effect on the liposome parameters were further statistically optimized by using BBD. The responses of all the experimental trials are summarized in Table 1. A quadratic model was selected based on the responses to analyse the experimental runs, and ANOVA was used to determine the response and influence on the independent variables.

**Table 1:** Observed responses in the Box–Behnken design during the optimization of independent variables involved in the preparation of liposomes.

Batches	Independent variables (coded)^#^			Dependent variables (coded)*	
	X_1_	X_2_	X_3_	Y_1_	Y_2_
LP-ASPM-F1	0	-1	+ 1	112.7 ± 2.9	55.75 ± 3.00
LP-ASPM-F2	+ 1	0	+ 1	118.6 ± 1.8	47.90 ± 2.6
LP-ASPM-F3	0	0	0	124.4 ± 0.4	48.07 ± 0.1
LP-ASPM-F4	0	-1	-1	132.3 ± 1.7	60.55 ± 1.6
LP-ASPM-F5	-1	0	-1	131.3 ± 0.8	59.23 ± 1.2
LP-ASPM-F6	0	+ 1	+ 1	116.9 ± 3.2	41.11 ± 1.1
LP-ASPM-F7	-1	+ 1	0	124.9 ± 2.1	52.01 ± 2.8
LP-ASPM-F8	-1	-1	0	131.3 ± 1.3	59.59 ± 1.5
LP-ASPM-F9	-1	0	+ 1	119.6 ± 1.5	57.44 ± 0.5
LP-ASPM-F10	0	+ 1	-1	112.5 ± 1.9	59.79 ± 2.2
LP-ASPMS-F11	+ 1	0	-1	113.3 ± 2.6	49.44 ± 1.3
LP-ASPM-F12	0	0	0	124 ± 0.3	48.96 ± 1.0
LP-ASPM-F13	+ 1	+ 1	0	118.4 ± 2.2	43.04 ± 0.9
LP-ASPM-F14	+ 1	-1	0	122.4 ± 2.5	46.20 ± 3.1
LP-ASPM-F15	0	0	0	124.3 ± 0.4	48.43 ± 0.5

^#^Independent variables: X_1_- Total lipid content, -1 = 100 mg, 0 = 150 mg, + 1 = 200 mg; X_2_- Sonication time, -1 = 5 min, 0 = 9 min, + 1 = 13 min; X_3_- Pulser on time, -1 = 6 min, 0 = 8 min, + 1 = 10 min. *Dependent variables: Y_1_- Particle size (nm); Y_2_—Drug EE (%EE).

#### Effect of independent variables on responses

- Particle size (Y_1_):

The particle size of the LPs varied from 112.5 ± 1.9 to 132.3 ± 1.7 nm, suggesting that the independent variables had a significant effect on the particle size of LP-ASPM. The ANOVA results (Supplementary information; Section S6b; Table S13) showed that the model was linear and significant since the *p* value for the model was 0.001, indicating that all the independent variables, viz*.,* total lipid content, sonication time and pulser “on time”, had a significant effect on the particle size. A decrease in particle size was observed with the increase in the independent variables, which was further evident in the Pareto (Supplementary Information; Figure S9), contour (Supplementary Information; Figure S10) and surface plots (Supplementary Information; Figure S11) for particle size. The polynomial regression equation generated for the effect of independent factors on the particle size is given below:

1 $$\begin{aligned} {\text{Particle size }}\left( {{\text{nm}}} \right){\text{ }} & = {\text{ 124}}.00 - {\text{ 4}}.{\text{65}}0{\text{X}}_{{\text{1}}} {-}{\text{ 3}}.{\text{112X}}_{{\text{2}}} \\ & \quad {-}{\text{ 3}}.{\text{412X}}_{{\text{3}}} + {\text{ 1}}.{\text{5}}00{\text{X}}_{{\text{1}}} {\text{X}}_{{\text{1}}} {-}{\text{ 1}}.{\text{675X}}_{{\text{2}}} {\text{X}}_{{\text{2}}} - {\text{ 5}}.{\text{425X}}_{{\text{3}}} {\text{X}}_{{\text{3}}} \\ & \quad + {\text{ }}0.{\text{175X}}_{{\text{1}}} {\text{X}}_{{\text{2}}} + {\text{ 4}}.{\text{225X}}_{{\text{1}}} {\text{X}}_{{\text{3}}} + {\text{ 6}}.{\text{65}}0{\text{X}}_{{\text{2}}} {\text{X}}_{{\text{3}}} \\ \end{aligned}$$

The effect analysis suggested an inversely proportional relationship between the independent variables and particle size, i.e., with the increase in all three independent variables, a significant reduction in the particle size was observed. The decrease in the particle size can be attributed to the fact that at longer sonication times, the cavitation effect on the particles increases for a sufficient period of time, thus resulting in random and uniform breaking of large liposome particles into small, thermodynamically stable liposome particles^48,49^. The reduction in particle size with respect to the increase in total lipid content could be due to the increased solubility of the phospholipids, which act as surfactants at the interface of the two phases^50^.

(b) Entrapment efficiency (Y_2_):

The %EE was found to be in the range of 41.11 ± 1.1% to 60.55 ± 1.6%, indicating a possible significant effect of the independent variables on the %EE of the LPs. The ANOVA results (Supplementary information; Section S6c; Table S14) showed that the model was linear and significant (*p* value: 0.014). The Pareto (Supplementary Information; Figure S12), contour (Supplementary Information; Figure S13), and surface plots (Supplementary Information; Figure S14) revealed that the %EE increased as the overall percentage of the independent variables decreased. The polynomial equation generated by the effect of independent variables on %EE is given below:

2 $$\ \begin{aligned} {\text{Entrapment efficiency }}\left( {\% {\text{EE}}} \right){\text{ }} & = {\text{ 48}}.{\text{14}} - {\text{ 5}}.{\text{511X}}_{{\text{1}}} {-}{\text{ 3}}.{\text{555X}}_{{\text{2}}} \\ & \quad {-}{\text{ 3}}.{\text{119X}}_{{\text{3}}} + {\text{ }}0.0{\text{8X}}_{{\text{1}}} {\text{X}}_{{\text{1}}} + {\text{ 1}}.{\text{17X}}_{{\text{2}}} {\text{X}}_{{\text{2}}} \\ & \quad + {\text{ 4}}.{\text{41X}}_{{\text{3}}} {\text{X}}_{{\text{3}}} + {\text{ 2}}.{\text{42X}}_{{\text{1}}} {\text{X}}_{{\text{2}}} \\ & \quad - {\text{ }}0.{\text{26X1X3 }}{-}{\text{ 4}}.0{\text{3X}}_{{\text{2}}} {\text{X}}_{{\text{3}}} \\ \end{aligned}$$

The entrapment efficiency decreased with increasing total lipid content, sonication time and pulser “on time”. The decrease in %EE with an increase in total lipid content may be because, at higher concentrations of lipids, the membrane stabilizing capacity of cholesterol decreases. This reduces the membrane fluidity and thereby decreases the amount of drug encapsulated in the bilayer^51^. The increase in %EE with decreasing sonication time can be attributed to the fact that at longer sonication times, the liposome particles tend to break into smaller particles, and during this stage, there is a chance of drug leakage from the vesicles. Moreover, a high sonication time further creates transient pores in the lipid bilayer of liposomes, resulting in drug leakage, thus reducing the %EE of the drug^52^. However, at a shorter sonication time, the particles may not break into smaller particles, and hence, there may be no or less drug leakage, which results in a higher %EE.

The model suggested an optimum solution consisting of 200 mg of total lipid content, 5 min of sonication time and 10 s of pulser “on time”, yielding a desirability value of 1.00. Experimental runs of the optimized solution were performed and compared with the predicted values from the regression model (Supplementary information; Section S6d; Table S15). The model was definite and suitable because the responses were within the 95% (α = 5%) prediction interval, indicating that the model had strong predictive power (Supplementary information; Section S6; Figure S15).

#### Effect of the amount of drug on the optimized ASPM liposomes

A linear dependency on the amount of ASPM incorporated into liposomes was observed. The results are given in Supplementary information; Table S16. The %EE increased as the concentration of ASPM increased to a plateau at high concentrations. Increasing the amount of drug did not significantly affect the particle size, PDI or ZP of the liposomes. However, precipitation of the drug was observed in the liposomes containing 20 mg and 25 mg of ASPM, which may be due to the saturation of the entrapping capacity of the liposomes. A total of 15 mg of ASPM was selected as the optimum amount of the drug to be used in the formulation of liposomes.

ASPM is a BCS-II drug that is lipophilic in nature; because of its lipophilicity, it interacts with and distributes itself within the lipidic bilayer of liposomes and thereby enhances the %EE. The increase in the %EE until reaching a plateau could also be due to the lipids used in the formulation of liposomes. The lipids impart stearic stabilizing effects in the liposomal colloidal system and prevent particle collision, resulting in the prevention of drug leakage. Hence, as the amount of drug increases, the lipids take up more of the drug, however, until the saturation level is reached. At the same time, cholesterol enhances the stability of liposomes, thereby preventing drug leakage, which further results in higher %EE values. Hence, a high drug-to-lipid ratio is one of the reasons for the increase in %EE up to a certain drug concentration in the formulation^53^.

#### Effect of different cryoprotectants on optimized ASPM liposomes

The particle size and PDI of the LP-ASPM prepared using mannitol and sucrose as cryoprotectants increased considerably compared with those of the respective unlyophilized LP. However, compared with those of unlyophilized LP, the particle size and PDI of LP-ASPM prepared using trehalose as a cryoprotectant were not substantially greater, and this finding has been corroborated by a previous report^54^. The less variation in liposome parameters with trehalose could be because of the ability of trehalose to form a high number of hydrogen bonds with water molecules, thereby forming a highly ordered cluster resulting in the disruption of bound water molecules^55^. Furthermore, the lower variation may also be the reason for the interaction of trehalose with DPPC. Trehalose is known to interact with DPPC and modify the symmetry of DPPC’s main transition, resulting in an increase in the T_m_ enthalpy and thereby increasing the packing density of DPPC polar head groups in the vesicle, resulting in increased liposome stability^56,57^. However, the cryoprotectants did not affect the ZP of the LPs. The results are given in supplementary information; Table S17.

#### Surface modification of LP-ASPM by EDC-NHS chemistry

The particle size and ZP of LP-DSPE-PEG decreased with increasing DSPE-PEG-COOH_2000_ concentration, which was in agreement with a previously published report^58^, and there was no effect on the PDI of the LPs (Supplementary information; Table S18). However, at 5% and 8% DSPE-PEG, the size was almost the same as that of non-PEGylated liposomes, and additionally, 5% DSPE-PEG resulted in comparatively lower PDI values with a sufficiently high ZP (> -30 mV) and was selected for the conjugation of liposomes. The steric repulsion between the PEG chains exposed from the outer layer of the liposomal bilayer results in a reduction in the particle size of the liposomes. The PEG chains on the liposome’s outer layer increase the vesicle curvature. A greater number of DSPE-PEG molecules are present on the outer surface of liposomes, and the addition of larger concentrations of DSPE-PEG-COOH_2000_ to liposomes further decreases the particle size of liposomes, which explains the observed reduction in particle size with increasing concentrations of PEG^59^. The decrease in ZP values (reduction in negative ZP values) could be due to the replacement of DPPC, a negatively charged lipid, with DSPE-PEG-COOH_2000,_ thereby decreasing the liposome surface charge.

### Characterization of unconjugated and conjugated ASPM liposomes

The average particle size, ZP and PDI of the conjugated and unconjugated liposomes are given in the Table S19 (Supplementary Information; Table S19). The conjugation of ligands onto the surface of LP-ASPM resulted in an increase in the particle size of the liposomes, possibly because of interactions between the ligands and the carboxylic group of DSPE-PEG-COOH_2000,_ suggesting successful conjugation of the ligands to the liposome surface^60^. Among the conjugated liposomes, the LP-PD-1 particles were larger because of the presence of the C_10_ lipid chain in PD-1, making them bulkier than the LP-PD-2 and LP-RGD particles. A substantial increase in the ZP (towards the positive side) was observed with LP-PD-1 and LP-PD-2 because of the presence of terminal positively charged arginine groups, which could have imparted a positive charge as they were conjugated onto the liposomal surface^61^. However, there were no changes observed in the ZP of the LP-RGD group. There was not much change observed in the PDI of the conjugated and unconjugated liposomes, suggesting a uniform dispersion of liposomes. The entrapment efficiency of the optimized LP‒ASPM was analysed by RP‒HPLC before and after conjugation. The results showed a slight decrease in the %EE of ASPM in the liposomes conjugated with PDs and RGD in comparison with the unconjugated liposomes. This decrease in %EE could be attributed to the longer stirring time and multiple steps with additional purification to remove the unconjugated ligands involved in the conjugation process, resulting in drug leakage and reduced %EE^35^.

**Fig. 1:**
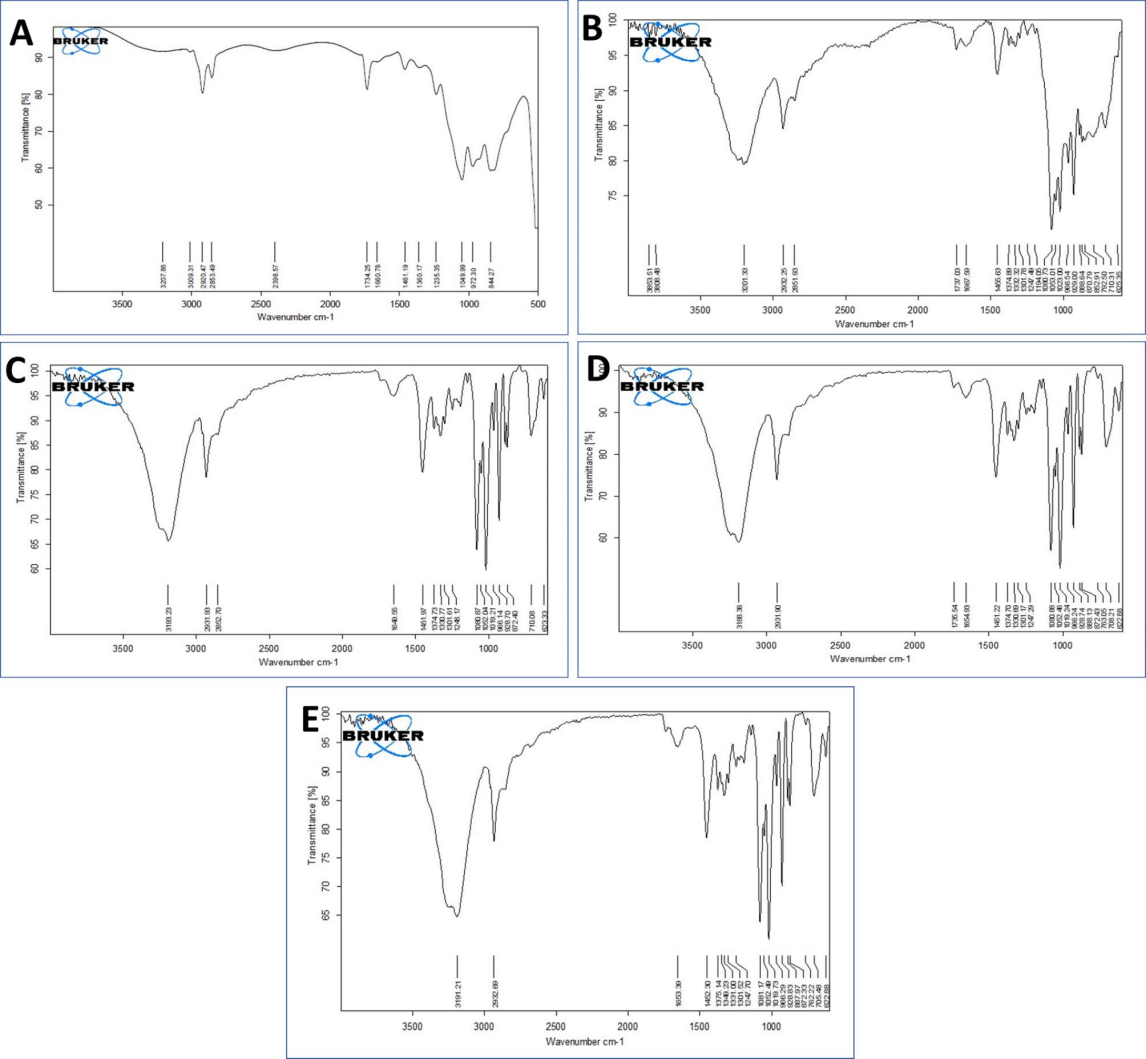
FTIR spectra of (**A**) LP-ASPM, (**B**) LP-DSPE-PEG, (**C**) LP-RGD, (**D**) LP-PD-1 and (**E**) LP-PD-2.

The FTIR spectrum of LP-ASPM (Fig. 1A) showed the peaks of ASPM as well as the excipients. Due to the presence of lipids, the peak size, shape and intensity of aromatic C=C bonds and aryl stretches (1660.71 cm^−1^) of ASPM were reduced. The peaks of the lipids used in the formulation of LP-ASPM are collectively observed in the range of 1400 cm^−1^ to 1600 cm^−1^. However, some of the characteristic peaks of ASPM (755.01 cm^−1^, 1092.87 cm^−1^, 1476.18 cm^−1^, 1700.04 cm^−1^ and 3034.09 cm^−1^) disappeared in the FT-IR spectrum of LP-ASPM, which suggested complete encapsulation of the drug into the liposomes, as reported in a previous study^26^. In the FTIR spectrum of LP-DSPE-PEG (Fig. 1B), a peak at 1737.03 cm^−1^ corresponding to the –C=O stretch of carboxylic acid and a peak at 2932.25 cm^−1^ corresponding to the –NH stretch was observed. The strong peak at 1737.03 cm^−1^ is almost negligible and absent in the FTIR spectrum of LP-ASPM (Fig. 1A), indicating the addition of DSPE-PEG-COOH_2000_ to the liposomes. The peak attributed to the -C = O orbital of carboxylic acid, which usually appears at 1730 cm^−1^, was observed to shift to 1649.55 cm^−1^ in the spectrum of LP-RGD (Fig. 1C). This shift in the wavenumber can be attributed to the presence of neighbouring bulkier groups. Although the peak attributed to the bidentate -NH_2_ group peak at 2931.93 cm^−1^ appears to be merged with the aliphatic region of the spectrum, a small peak can be observed, indicating the presence of amide bonds. The FTIR spectra of both the LP-PD-1 and LP-PD-2 liposomes (Fig. 1D,E, respectively) showed almost the same peaks as those of the LP-RGD liposomes. The presence of both –C=O and NH_2_ peaks in all the spectra of the conjugated liposomes confirmed the successful conjugation of the ligands to the liposomes via the linker DSPE-PEG-COOH_2000_ by the formation of amide (CONH) bonds.

**Fig. 2:**
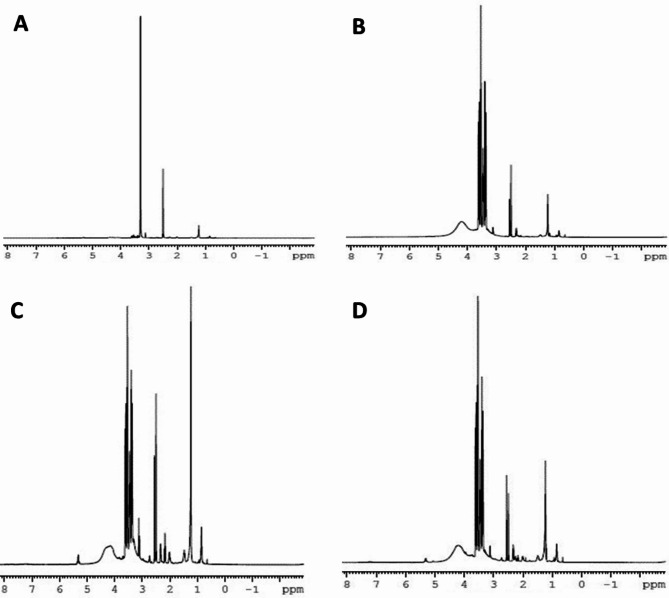
^1^H-NMR spectra of (**A**) LP-DSPE-PEG, (**B**) LP-RGD, (**C**) LP-PD-1 and (**D**) LP-PD-2.

It is quite challenging to completely understand and decode the presence of expected protons in the proton NMR spectrum of conjugated liposomes due to the presence of several components, such as drugs, ligands and lipids in the formulation. Furthermore, the amount of PDs and RGD used in the formulation was quite low; therefore, the characteristic peaks corresponding to the ligands may not be readily observed in the NMR spectrum. In the NMR spectrum of LP-DSPE-PEG (Fig. 2A), the peaks observed at 0.838–1.23 ppm corresponds to the terminal methyl (-CH_3_) and methylene (-CH_2_) protons of DSPE. A characteristic chemical shift at 3.623 ppm was observed which corresponds to the highly de shielded α-protons of the repeated units of ether (-CH_2_-CH_2_-O) links of PEG chain^62^. The NMR spectra of all the conjugated liposomes (Fig. 2B–D) showed broadened peaks between 3 and 5 ppm, which is mainly due to an increase in the electron density as a result of the conjugation reaction. Notably, the signal peak at 3.623 ppm corresponding to PEG chain was also observed in the NMR spectrum of LP-RGD (Fig. 2B). A peak at 1.49 ppm indicated the presence of methylene groups in the arginine side chain. In the case of LP-PD-1 (Fig. 2C), the peak observed at 1.236 ppm corresponds to the two methyl groups of the leucine side chain, while the methylene groups of the C_10_ chain attached to the lysine side chain of PD-1 resonated at a signal peak at 1.460 ppm, with a terminal methyl peak at 0.871 ppm. The NMR spectrum of LP-PD-2 (Fig. 2D) exhibited all the expected peaks of the amino acids present in PD-2. A weak signal was observed at 5.31 ppm in the spectra of both LP-PD-1 and LP-PD-2, which is likely due to the amide N–H proton signal from the conjugation reaction. The downfield shift of the amide protons could be attributed to the complex environments of conjugated liposomes, where the liposomal bilayer may shield the amide bond resulting the observed shift. Additionally, the primary amine group of glycine in all the ligand-conjugated liposomes (LP-RGD, LP-PD-1 and LP-PD-2) was converted into a broad peak due to interactions with the lipids of the liposomes during the formation of amide bonds. Thus, suggesting a successful conjugation of the ligands to the liposomes.

The overlay thermograms of pure ASPM and the developed liposomal formulations are shown in Supplementary information; Figure S16. The DSC thermogram of the pure ASPM showed a sharp and intense endothermic peak at 146.92 °C, corresponding to the melting point of ASPM, also suggesting the crystalline nature of the pure drug^20^. The DSC thermograms of LP-ASPM, LP-PD-1 and LP-PD-2 showed drug peaks at 143.59 °C, 137.94 °C and 130.67 °C, respectively. The DSC thermogram of LP-ASPM showed an endothermic peak at 160 °C, which could be attributed to cholesterol (148 °C). The thermotropic changes in the peak temperature, which alters the structural conformation of cholesterol, could be the reason for this shift in the melting point of cholesterol^63^. The broad endothermic peak observed in the thermogram of LP-ASPM at 230 °C could be attributed to SPC (236.1 °C). The peaks observed in the range of 200–230 °C in the thermograms of LP-PD-1 and LP-PD-2 correspond to the amino acids present in the peptide dendrimers. The peak broadening observed in the thermograms of the liposome formulation suggested the loss of crystallinity of ASPM, indicating that the drug was amorphized within the liposomes^35^. The endothermic peaks of these formulations were observed with a broader melting transition, reduced peak intensity and a shift in the melting points. These changes in the thermograms could be because of the dissolution of ASPM in the phospholipid matrix at higher temperatures and the amorphization of the drug, suggesting that ASPM is available in a molecularly dispersed state in the formulations^64^.

**Fig. 3:**
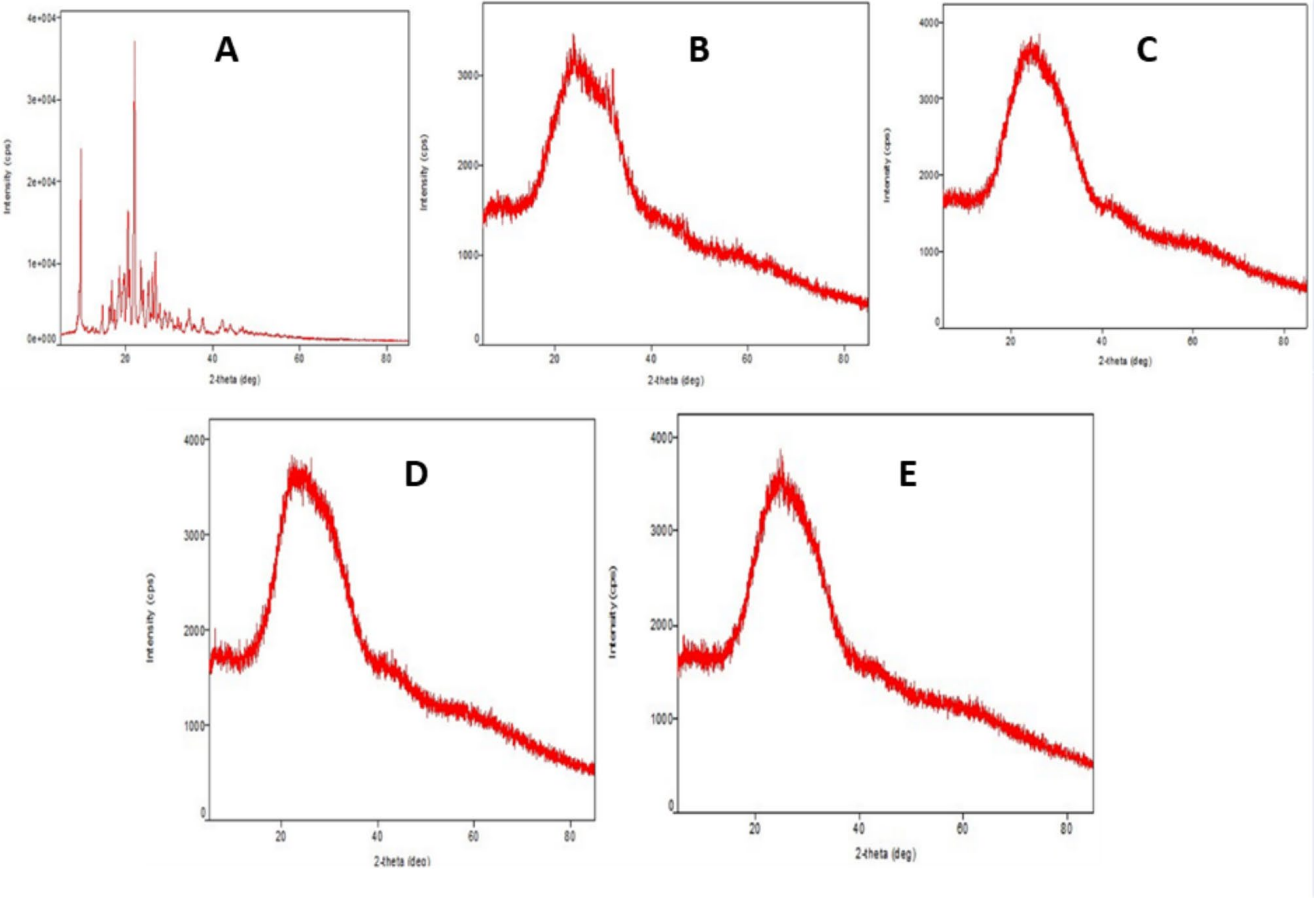
X-ray diffractograms of (**A**) ASPM, (**B**) LP-ASPM, (**C**) LP-RGD, (**D**) LP-PD-1 and (**E**) LP-PD-2.

X-ray diffractograms of plain ASPM, LP-ASPM, LP-RGD, LP-PD-1 and LP-PD-2 are shown in Fig. 3. The XRD pattern of plain ASPM (Fig. 3A) showed two intense peaks, one at a 2ɵ value of 9.70° and the other at 22.05°^65^. The XRD pattern of LP-ASPM (Fig. 3B) revealed a very small but sharp peak at a 2ɵ value of 25.61°, which could be due to the presence of ASPM, and the reduced intensity suggested possible amorphization of the drug into the liposomes. The XRD results of the conjugated liposomes (Fig. 3C,D,E) showed similar patterns with no intense peaks of ASPM, clearly indicating the possible amorphization of the entrapped ASPM in the liposomes.

**Fig. 4:**
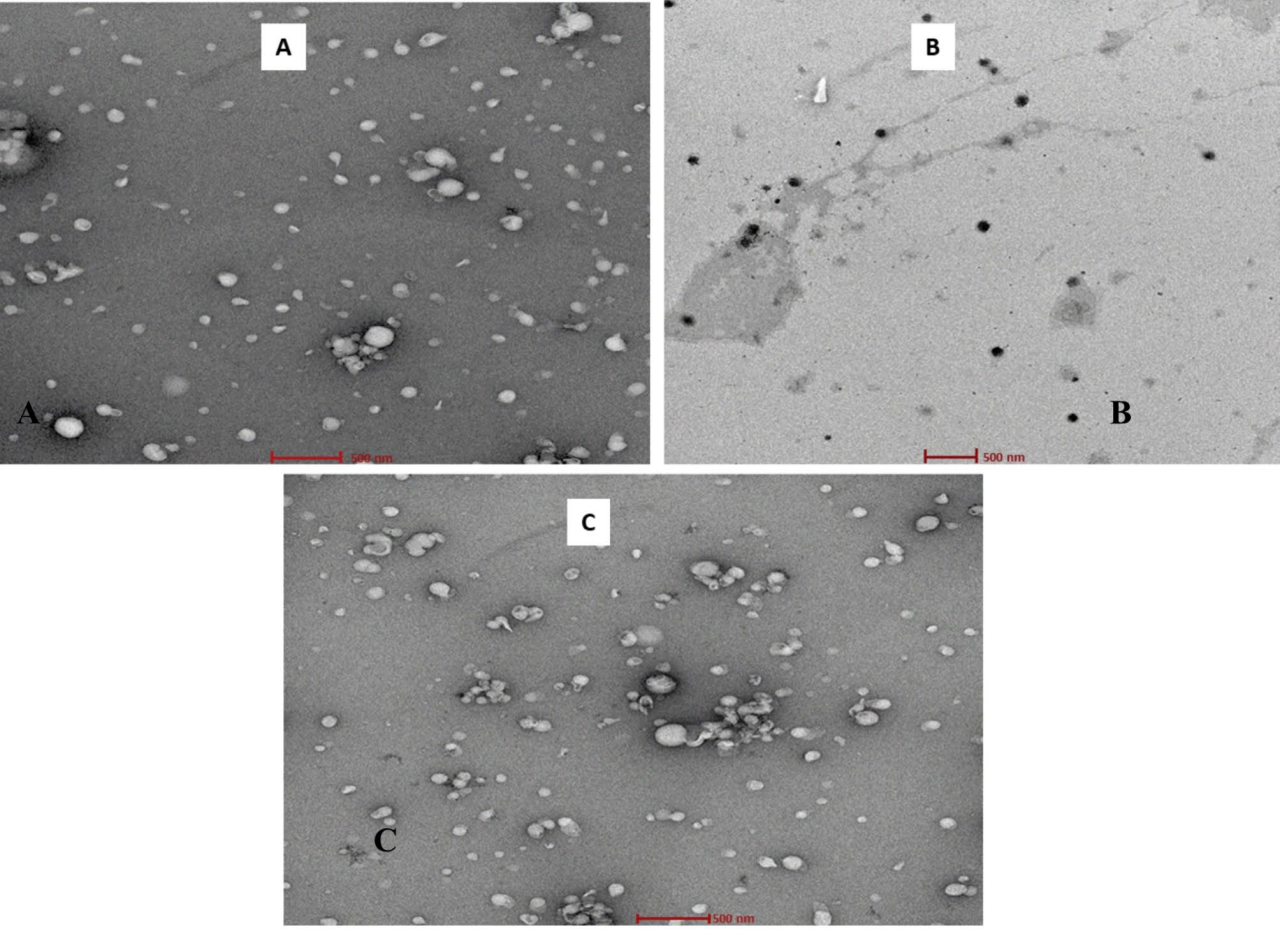
TEM images of (**A**) LP-ASPM, (**B**) LP-PD and (**C**) LP-RGD.

TEM images of both conjugated and unconjugated liposomes (Fig. 4) revealed monodisperse, well-separated vesicles of approximately 100 nm with a discrete spherical shape. The TEM image of LP-ASPM (Fig. 4A) showed no layer around the particles. However, the images of the conjugated liposomes (Fig. 4B,C) showed a distinct layer around the particles. This distinct dark halo surrounding the liposomes is mainly due to PEGylation, indicating that conjugation has taken place and that the liposomes are anchored with ligands. The dark layer corresponds to the electron-dense PEG chains, as the presence of PEG chains increases the electron density around the liposomes^66^. The dark layers appearing in the TEM images of the conjugated liposomes were more consistent and continuous, suggesting a homogeneous and uniform distribution of PEG chains around the liposomes. The uniform distribution also suggested a higher density of PEGylation^67^.

**Fig. 5:**
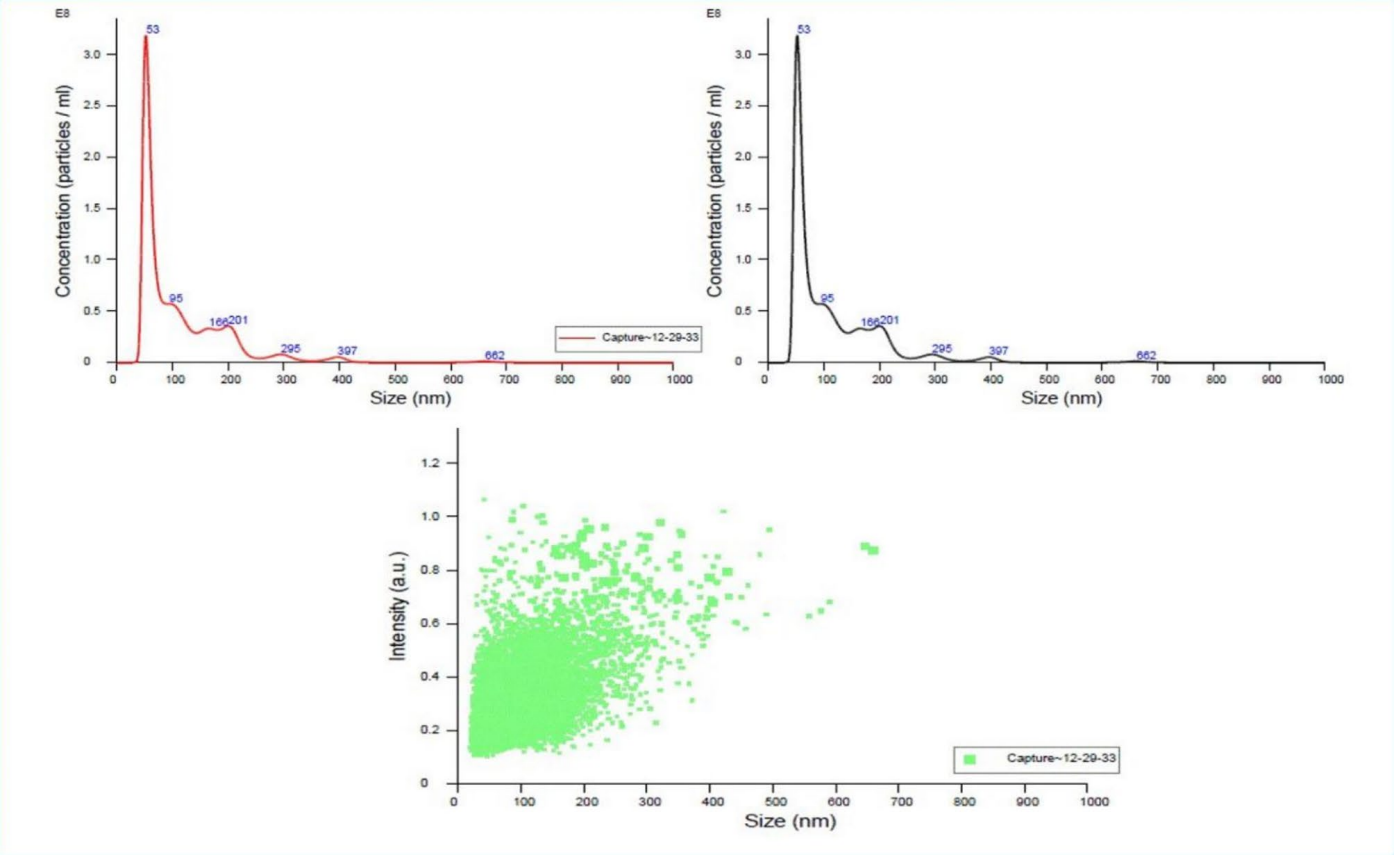
NTA images of the optimized LP‒ASPM. (**A**) FTLA concentration, (**B**) average FTLA concentration and (**C**) size/intensity.

The NTA results for LP-ASPM are shown in Fig. 5. A single peak of liposomes with an average size of 110.1 nm was reported. The average D10, D50 and D90 values were 48.8 nm, 77.3 nm and 207.1 nm, respectively. The concentration of the vesicles in the optimized LP‒ASPM dispersion was found to be 1.29 × 10^13^ particles/ml. The observation from the NTA suggested that the particle size obtained from the NTA nearly corroborated the particle size obtained from DLS.

### In vitro drug release studies

**Fig. 6:**
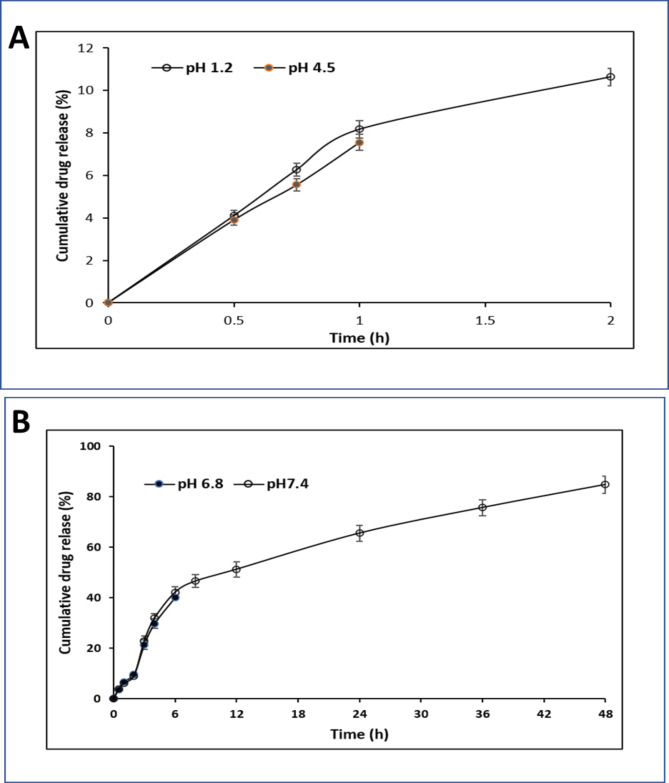
In vitro drug release study of ASPM from ASPM liposomes. (**A**) Release studies in HCl (pH 1.2) and ammonium acetate buffer (pH 4.5). (**B**) Release studies in phosphate buffer (pH 6.8) and phosphate buffer (pH 7.4).

An in vitro drug release study was performed using the dialysis sac method. For the pure ASPM dispersion, the literature reports, rapid drug diffusion through the dialysis membrane in gastric fluid (pH 1.2), followed by a release of more than 90% in intestinal fluid (phosphate buffer pH 6.8) within 180 min, aligning with the expected outcomes^24^. LP-ASPM showed 10.64 ± 0.41% ASPM release in a pH 1.2 HCl solution in the first 2 h, and 7.56 ± 0.36% of the drug was released within 1 h in a pH 4.5 ammonium acetate buffer (Fig. 6A). The decrease in drug release at acidic pH can be attributed to the pH-dependent increase in the solubility of ASPM. At lower pH, such as at pH 1.2 and pH 4.5, the acidic environment leads to protonation of the drug molecule, resulting in the precipitation of the drug within liposomes, thereby reducing the solubility and subsequent release of the drug. Furthermore, highly acidic pH conditions lead to destabilization of the liposome structure, altering the release kinetics of the drug^68^. In pH 6.8 phosphate buffer, 40.19 ± 1.89% of the drug was released in 6 h, while in pH 7.4 phosphate buffer, 84.8 ± 3.5% of the drug was released in 48 h (Fig. 6B). ASPM was observed to be released continuously but in a sustained manner at pH 7.4, as the liposomal formulation had a greater solubilizing effect on the drug at pH 7.4 and thus increased the release rate of ASPM^69^. The sustained release can be attributed to the encapsulation of ASPM in the lipid membrane, which is held within a small fragment of the liposomal membrane from which the drug is released slowly through diffusion and dissolution from the lipid bilayer^70^. The obtained results are in agreement with previously reported studies^22,65^.

### In vitro cell viability studies

**Fig. 7:**
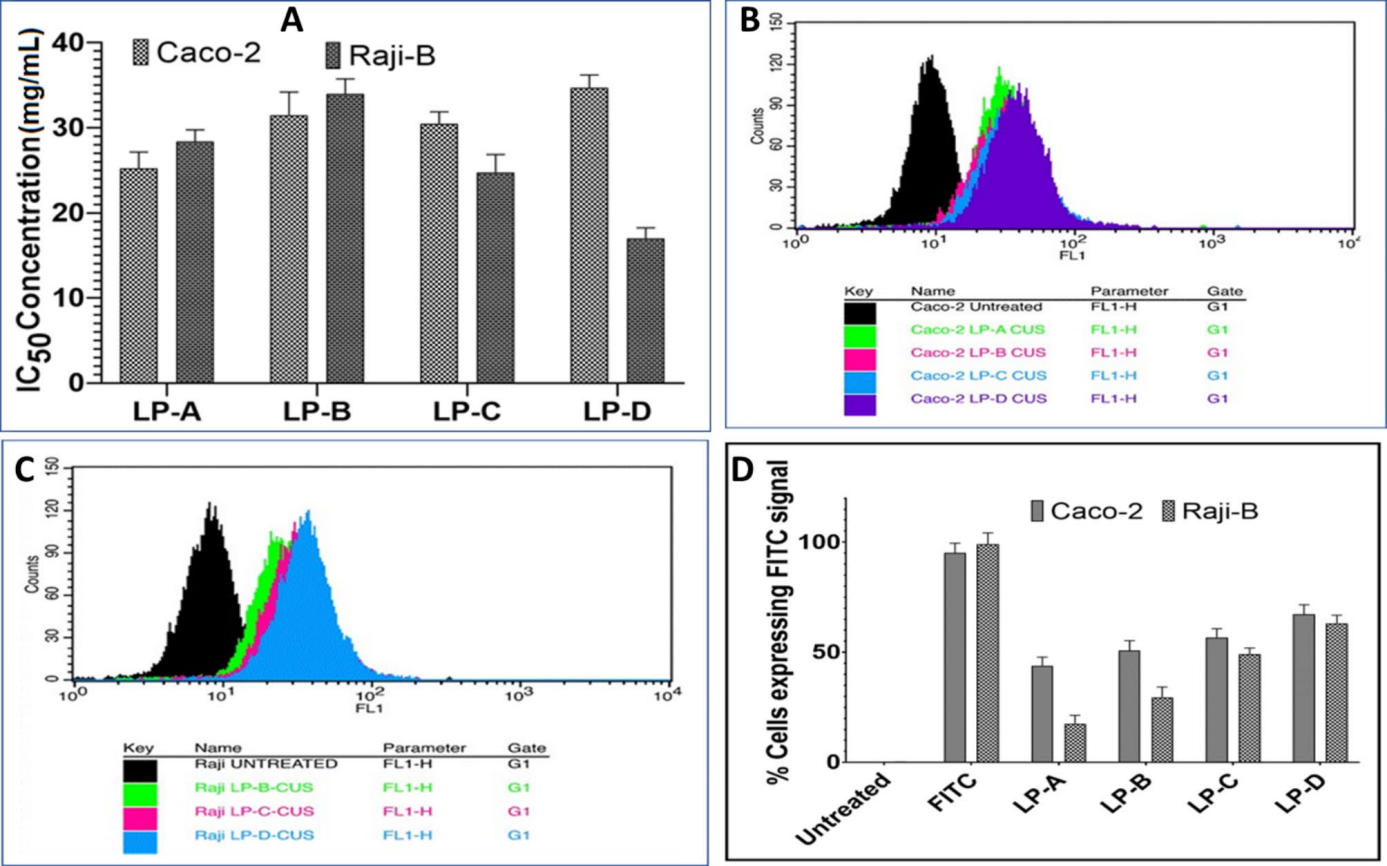
Cellular studies of liposomal formulations. (**A**) IC_50_ values of liposomal formulations on Raji B cells and Caco2 cells. FACS analysis of cellular internalization of liposomal formulations (**B**) in Caco2 cells and (**C**) in Raji B cells. (**D**) Cellular internalization of the liposomal formulation in Caco-2 and Raji-B cells. LP-A: Blank liposomes; LP-B: Liposomes + RGD; LP-C: Liposomes + PD-1 (lipidated peptide dendrimers) and LP-D: Liposomes + PD-2 (nonlipidated peptide dendrimer).

In vitro cell viability studies were performed on both Raji-B and Caco2 cells to assess the biocompatibility of the developed formulation. The cytotoxicity profile of the standard drug (ASPM) has already been extensively well established in the previous studies conducted by our research team. These studies demonstrated that ASPM is a non-cytotoxic drug with more than 80% cells remaining viable even at the highest concentration of ASPM tested in both Caco-2 and Raji –B cells^21,22,71^. Blank-Liposome (LP-A), LP-ASPM (LP-B), LP-RGD (LP-C), LP-PD-1 (LP-D) and LP-PD-2 (LP-E) did not show any significant cytotoxicity against Raji B cells or Caco2 cells in the experimental concentration range of 0.312 µg/mL to 10 µg/mL with approximately 80% cell viability after 24 h. Approximate IC50 values for Raji B cells (Fig. 7A) were observed at 28.36 µg/mL, 33.94 µg/mL, 24.75 µg/mL, 17.03 µg/mL and 19.56 µg/mL for LP-A, LP-B, LP-C, L-D and LP-E, respectively. In Caco-2 cells, IC_50_ values of 25.24 µg/mL, 31.49 µg/mL, 30.47 µg/mL, 34.70 µg/mL and 32.66 µg/mL, respectively, were detected (Fig. 7A). One-way ANOVA and Bartlett’s test further confirmed the absence of any significant change in cell viability at different concentrations among the different formulations. The 10 µg/mL concentration of all the compounds was selected for the uptake studies since there was no significant cytotoxic effect on Raji B and Caco2 cells. Approximately 80% cell viability was observed for both Caco2 and Rajji B cells, indicating the biocompatible nature of the developed liposomal formulation both with and without surface modification. Cytotoxicity studies of the two PD (PD-1 and PD-2)-conjugated liposomes revealed similar results. The PEGylation of liposomes is known to reduce their cytotoxicity and enhance their biocompatibility. Despite having a positive charge, PDs are less cytotoxic^72,73^, which was also confirmed by the current study. The cytotoxicity of the PDs is further reduced by the presence of PEG chains, as they shield the positive charge on the PD surface^74^. The low toxicity of the PDs can also be attributed to the fact that these PDs can degrade into biocompatible amino acids, which constitute important building blocks of living systems^75^.

### Cellular uptake studies

The cell uptake study was performed on both Caco2 and Raji-B cells for all the liposomal formulations to study the behaviour and interaction of liposomes with cells and also to understand the effect of surface modification on liposomal uptake. The obtained results suggested a significant difference in the uptake percentage of the formulation. The percentage uptake of unmodified liposomes (LP-A) was 43.93 ± 3.1%, while that of conjugated liposomes (LP-B (LP-RGD), LP-C (LP-PD-1) and LP-D- (LP-PD-2)) was 50.78 ± 3.8%, 56.75 ± 3.2% and 67.34 ± 4.1%, respectively, in Caco-2 cells, while in Raji B cells, the percentage uptake of LP-A, LP-B, LP-C and LP-D was 19.55 ± 3.5%, 29.55 ± 3.2%, 49.22 ± 4.7% and 69.23 ± 4.2%, respectively. The enhanced internalization of the liposomal formulation in Caco-2 and Raji-B cells was observed with an increase in the FL1 value for the surface-modified liposomes compared to that of the unmodified and control liposomes (Fig. 7B,C). The uptake of LP-D (liposome conjugated with nonlipidated peptide dendrimer, PD-2) was greatest in both Caco-2 and Raji B cells, but the percentage was greater in Raji B cells than in Caco-2 cells, which can be attributed to the greater affinity of LP-D for M cell-mediated uptake.

The increase in the cellular uptake of the modified liposomes compared with that of the unmodified liposomes could be because the functional groups on the surface of the modified liposomes directly or indirectly helped to enhance the internalization of the liposomes. The reason for the enhanced uptake of LP-B (LP-RGD) could be that the RGD moiety on liposomes acts as a ligand that specifically binds to integrin receptors that are overexpressed on the apical side of M-cells^76,77^. PDs reportedly enhance the uptake of drugs via enterocytes and Peyer’s patches in the small intestine^78^, which could be the reason for the enhanced uptake of PD-conjugated liposomes (LP-C and LP-D) in comparison with that of RGD-conjugated liposomes. The presence of positively charged arginine residues in PDs can enhance cellular uptake through ionic interactions with negatively charged phosphopeptides and/or phospholipids in the cell membrane^79^, and the guanidine functional group of arginine forms a hydrogen bond with the phospholipids of the cell membrane bilayer and thus helps in the internalization of the nanocarrier, indicating the ability of arginine-rich peptides to translocate across the cell membrane^80,81^. Furthermore, lysine present in PD interacts with the anionic cell membrane by destabilizing it and ingressing into the cell membrane, thus improving cell permeation^82^. The decreased cellular uptake of LP-C (LP-PD-1) compared to LP-D (LP-PD-2) could be because of the hydrophobic interactions of the decanoic acid lipid chain attached to the peptide dendrimer, leading to the aggregation of the lipidated PDs and resulting in an increase in the size of the PD, which could affect the intestinal absorption of the lipidated PDs^83^. Furthermore, the chain length of the lipid attached to the PD increases the size of the PD, rendering it difficult for it to be taken up by the intestinal epithelium^84^, which was also observed in previously reported studies^78,85^. The uptake of unmodified liposomes (LP-A) was greater in Caco2 (Fig. 7D) cells because the lipids in the liposomes open tight junctions between adjacent epithelial cells and thereby enhance permeation and uptake across Caco2 cells^86^. The translocation of unmodified liposomes across Raji B cells decreases and strongly depends upon M cell-based phagocytosis^87^ because of which the internalization of LP-A was less than that of LP-B, and LP-C/ LP-D was high (Fig. 7D).

### Mechanism of cellular uptake

In the present study, an attempt was made to understand the uptake pathway of various surface-modified liposomes in Raji B and Caco2 cells using a cellular poisoning study. The cellular uptake of the control (cells incubated without poisons) was considered 100%, and the subsequent decrease in cellular uptake with respect to the control was calculated to determine the cellular uptake pathway of the liposomes. The obtained results suggest that the majority of liposomal nanoparticles were internalized in the control group, while the percentage of liposomes taken up decreased with different levels of uptake poisoning, suggesting the possible role of various internalization pathways.

**Fig. 8:**
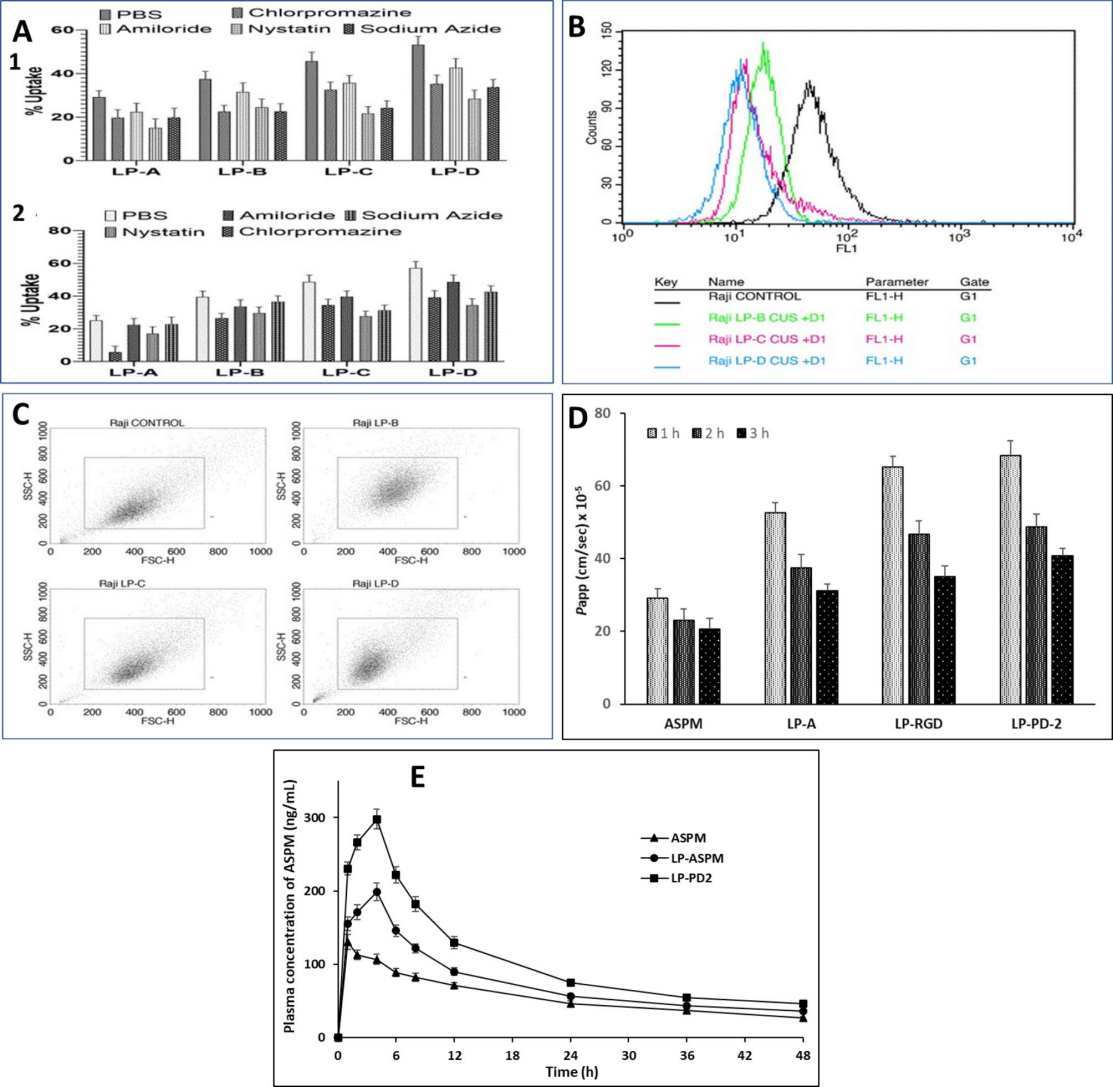
Results of cell uptake studies, FACS analysis, Scatter plot, permeability studies and pharmacokinetics studies of different liposome formulations. (**A**) Mechanism of cell uptake of different liposome formulations in (1) Raji B cells and (2) Caco2 cells. (**B**) FACS analysis of the effect of receptor saturation on the cellular uptake of liposomal formulations. (**C**) Scatter plot demonstrating the effect of receptor saturation on the cellular uptake of surface-modified liposomes by Raji-B cells. (**D**) Permeability of free ASPM, LP-ASPM, LP-RGD and LP-PD-2 through the everted rat ileum and (**E**) Plasma concentration *vs*. time profile of plain ASPM, LP-ASPM liposomes and LP-PD-2 liposomes.

In the case of the blank liposomes (LP-A), the effect of the uptake poisons in the cellular uptake studies of Raji-B cells was not very significant. However, a decrease in uptake was observed in the chlorpromazine (19.7 ± 3.7%) and nystatin (15.1 ± 4.1%) incubation groups, suggesting that the cellular uptake of LP-A was predominantly clathrin- and caveolae-based endocytosis. The variation in the cellular uptake of LP-RGD (LP-B) compared with that of LP-A in the case of the control (37.5%), chlorpromazine (22.5 ± 2.9%) and nystatin (24.6 ± 3.7%) groups indicates a significantly greater uptake of LP-B than of LP-A, which could be because of the presence of surface ligands with charge-based affinity towards the cell surface. In the case of LP-A and LP-B, the sodium azide (17.4 ± 2.7%; 22.6 ± 3.6%, respectively) poisoned group showed a higher decrease in uptake compared to LP-A, suggesting that LP-B is predominantly taken up by energy-dependent endocytosis, which could be attributed to the surface groups (RGD ligands) present on the liposomes. The uptake poisoning results for LP-C (LP-PD-1) and LP-D (LP-PD-2) were similar. A significant difference was observed with respect to Nystatin (LP-C: 21.7 ± 3.1%; LP-D: 28.5 ± 3.9%) and sodium azide (LP-C: 24.3 ± 3.2%; LP-D: 33.7 ± 3.6%), which suggests that cellular uptake occurs mainly by energy-mediated endocytosis and caveolae-based endocytosis (Fig. 8A1). The surface groups on liposomes conjugated with PDs contain amino acid residues that can bind to αvβ3 receptors, which facilitates the energy-dependent uptake of liposomes conjugated with PDs. The decrease in cellular uptake in Caco2 cells (Fig. 8A2) demonstrated a similar pattern for different uptake blockers as that observed in the case of Raji-B cells, except that there was no significant effect on energy-dependent endocytosis, unlike in Raji-B cells, which can be attributed to the absence of specific receptors involved in the active transport of dendrimer-modified liposomes.

### Receptor saturation study

In the receptor saturation study, the receptor was saturated with ligand before initiation of the uptake study. The saturation with the ligands blocks the receptor due to receptor saturation, and hence, the surface-modified formulation would not show any internalization via a ligand-mediated pathway. The % uptake in the unsaturated group was taken as 100%, and the obtained results suggested a significant decrease in the uptake of liposomes after receptor saturation using RGD and PDs (Fig. 8B). The FACS analysis of receptor saturation study (Fig. 8B) demonstrated a leftward shift in the fluorescence intensity confirming the reduced capacity of the cells to internalize liposomes after saturation with ligands. Compared with that of the untreated group, the % decrease in the cellular uptake of liposomes was 40.84 ± 4.2%, 69.46 ± 4.9% and 85.96 ± 3.7% for LP-B, LP-C and LP-D, respectively. The greatest suppression of cellular uptake was found for LP-D (LP-PD-2) as the receptor saturation limits the internalization efficacy of LP-D by restricting the number of available binding sites. This resulted in a negligible uptake of the LP-D, which was facilitated by PD-2 via the receptor-mediated pathway, and hence there was a decrease in uptake after receptor saturation. The suppression of internalization after receptor saturation was greater for LP-C (LP-PD-1) than for LP-B (LP-RGD), suggesting that the internalization efficiency of dendrimer-modified liposomes was greater than that of RGD-modified liposomes. This indicates that the LP-C possess a higher internalization efficiency prior to receptor saturation, whereas LP-B showed relatively low internalization efficiency but greater resistance to saturation-dependent suppression. The effect of receptor saturation on all the surface-modified liposomal formulations could be due to the presence of amino acids in the peptide dendrimer, and RGD (used as a substrate for receptor saturation) blocks the receptor in a similar manner to RGD or another peptide dendrimer, which facilitates uptake by binding to the M cell receptor. It can be clearly observed from the scatter plots (Fig. 8C) that the intensity of the liposomal formulation decreased in the sequence of LP-D > LP-C > LP-B. The observed sequence provides insight into the effect of receptor saturation on cellular internalization. In the case of LP-D, the intensity plot shifted towards the origin with a decrease in the scattered area limited between 200 and 400. This suggests a more efficient cellular uptake, which is likely due to the more optimal receptor-ligand interaction that enhances the internalization. The smaller scatter area indicates a more uniform and effective interaction, which may be due to the availability of fewer receptors for binding. In the case of LP-C, the intensity was distributed between 400 and 600, showing a broader range of internalization compared to LP-D. This suggests that the effect of the surface-modifying agent on cellular internalization was greater for LP-D than for LP-C. Furthermore, the wider scattering of data points for LP-C indicates more variability in internalization, which could be because some of the cells experience lower uptake due to less receptor saturation. In the case of LP-B, the intensity was scattered between 200 and 600, but the area was greater than that of LP-C. The greater scatter area reflects that the formulation may interact less efficiently with the cell surface, indicating the possibility of decreased binding and internalization. The large scattering area suggests that RGD is less efficient at facilitating cellular internalization than the synthesized PDs.

### Animal studies

#### Ex vivo drug permeation studies

The permeation of the free drug (ASPM), LP-ASPM, LP-RGD and LP-PD-2 was evaluated across the everted ileum sac for a period of 3 h (Fig. 8D). LP-ASPM showed a considerable increase in the *P*app value (1 h: 52.6 ± 2.8 × 10^–5^ cm/s; 2 h: 37.5 ± 3.6 × 10^–5^ cm/s; 3 h: 31.1 ± 1.9 × 10^–5^ cm/s) compared to that of the free drug (1 h: 29.1 ± 2.6 × 10^–5^ cm/s; 2 h: 23.1 ± 3.1 × 10^–5^ cm/s; 3 h: 20.6 ± 3.0 × 10^–5^ cm/s). This greater permeability of liposomes may be attributed to their lipidic nature and the interaction of phospholipid head groups with mucus glycoproteins, which might have resulted in restructuring of the mucous membrane^88^. LP-RGD (1 h: 65.1 ± 3.1 × 10^–5^ cm/s; 2 h: 46.6 ± 3.8 × 10^–5^ cm/s; 3 h: 35.1 ± 2.9 × 10^–5^ cm/s) and LP-PD-2 (1 h: 68.4 ± 3.9 × 10^–5^ cm/s; 2 h: 48.7 ± 3.6 × 10^–5^ cm/s; 3 h: 40.8 ± 2.1 × 10^–5^ cm/s) showed enhanced permeation through the everted ileum sac compared to LP-ASPM. This could be attributed to the RGD-M integrin interaction and peptide dendrimer-intestinal membrane interaction with the conjugated liposomes. LP-PD-2 showed greater permeation than LP-RGD, which was also supported by the cell uptake studies.

#### Pharmacokinetic studies

The plasma concentration *vs*. time profile of ASPM in different forms is given in Fig. 8E. The pharmacokinetic parameters are given in the Table S20 (Supplementary Information; Table S20). The extent of absorption of the drug (AUC_0-t_) was considerably greater for all the ASPM liposomal formulations than for the plain ASPM. The AUC_0-t_ (3602.53 ± 331.67 h*ng/mL) of LP-ASPM was significantly greater than that of plain ASPM (2658.63 ± 220.89 h*ng/mL). The increase in the relative bioavailability of the liposomal formulation can be attributed to the nanoscale size of the liposomes, which are effectively taken up by the lymphoid tissue, thereby preventing first-pass hepatic metabolism^89^. The small particle size of liposomes provides a larger surface area, aiding in the diffusion of the drug across the intestinal epithelium and providing a high concentration gradient, resulting in an increased rate of drug absorption^24^. Furthermore, the lipids of liposomes stimulate the production of chylomicrons, and the drugs present in liposomes are associated with chylomicrons, forming a large lipid micellar structure^90^. These chylomicrons are taken up by the lymphatic circulation and are then released into the systemic circulation, thereby bypassing first-pass hepatic metabolism and enhancing the bioavailability of ASPM^91^. The AUC_0-t_ of the conjugated (LP-PD-2) liposomes (5081.24 ± 378.66 h*ng/mL) significantly improved, indicating that the oral bioavailability of the conjugated liposomes (LP-PD-2) was greater than that of the plain ASPM and unconjugated (LP-ASPM) liposomes. The enhanced bioavailability of LP-PD-2 is mainly due to the presence of PD on the liposomal surface. Owing to their structure, PDs interact with enterocytes and with the β1 integrins present on the M-cells of the Peyer patches of the small intestine and facilitate the uptake of liposomes, thereby increasing the rate of absorption, which in turn increases the bioavailability of the drug^78^. The maximum plasma concentration (Cmax) of plain ASPM was 130.66 ± 7.01 ng/mL. The Cmax value was ~ 1.54 times greater for LP-ASPM (198.68 ± 12.37) than for plain-ASPM. On the other hand, an overall improvement in the Cmax of LP-PD-2 (297.89 ± 16.67) was observed in comparison with plain ASPM and LP-ASPM. An approximately 1.49-fold increase in the Cmax was observed in the LP-PD-2 group compared to the LP-ASPM group. The Tmax was found to be 1 h for the plain drug, whereas the Tmax was delayed by up to 4 h for both LP-ASPM and LP-PD-2. The half-life (t_1/2_) of plain ASPM was found to be 25.2 ± 2.15 h, and the t_1/2_ of LP-ASPM was found to be 44.48 ± 3.26 h, indicating a ≈ 1.7-fold greater increase in the half-life of ASPM than that of the plain drug. However, the t_1/2_ values of LP-PD-2 were 55.91 ± 3.89 h and ≈1.2 times greater than the half-life of LP-ASPM. The considerable increase in the t_1/2_ of LP-PD-2 is mainly due to the PEGylation of liposomes, which is known to increase the circulation half-life, reduce renal clearance and improve biological activity. The elimination rate constant _(Kels_) for LP-ASPM and LP-PD-2 were 0.016 ± 0.001 h^−1^ and 0.012 ± 0.001 h^−1^, respectively, in comparison with the K_el_ of plain ASPM (0.0271 ± 0.002 h^−1^). The mean residence time (MRT) of LP-PD-2 (64.03 ± 4.78) was significantly greater than that of LP-ASPM (54.18 ± 4.16) and plain ASPM (36.00 ± 3.16). As supported by previous reports, the conjugation of PD-2 to LP-ASPM reduces drug release, leading to slower elimination of ASPM from the system^35^. Furthermore, the cellular uptake of LP-PD-2 is enhanced due to the targeting ability of PD-2 and the interaction of surface-charged PDs with lipid membranes, which could reduce the elimination rate of the drug from systemic circulation^92,93^. An increased MRT suggests that the drug remains in the bloodstream for an extended period when administered as liposomes. Additionally, PEGylation of liposomes increases the MRT, as PEG is known to prolong drug circulation. A decrease in the elimination rate of LP-PD-2 results in an increase in the residence time of the drug in the body, as evidenced by the obtained results. An increase in the MRT of LP-PD-2 leads to greater drug accumulation at the target site, improving therapeutic efficacy. The obtained pharmacokinetic results are characteristic of conjugated liposomal drug delivery systems, which improve therapeutic efficacy by providing sustained drug release and reduced toxicity^94^. The results of pharmacokinetic studies were in sync with those of in vitro cell uptake studies, where LP-PD-2 showed the highest permeation and uptake across Caco2 and Raji-B cells, and similar results were also reported in a previous study^23^.

#### Pharmacodynamic study

Pharmacodynamic studies were performed using a psychostimulant-induced hyperactivity model in rats. Normal rats exhibited locomotor counts of 367 ± 38, 401 ± 39 and 382 ± 35 at 1, 2 and 3 h, respectively. Intraperitoneal administration of L-DOPA and carbidopa induced hyper locomotor activity in the rats, as indicated by significantly greater locomotor counts than in the normal rats (Table 2). ASPM (5 mg/kg) reduced locomotor counts to less than the normal values at all the time points tested. All the rats administered ASPM liposomes showed greater reductions in locomotor counts than those administered with the ASPM solution. Among the liposomes, the liposomes containing PD-2 (LP-PD-2) showed the greatest reduction in locomotor count, which could be due to the effective absorption of ASPM from LP-PD-2. These observations further support the results obtained in cell line studies where LP-PD-2 showed better cellular uptake. This large reduction in the LMA may serve as an excellent antipsychotic treatment for treating schizophrenia and bipolar disorder.

**Table 2:** Locomotor counts of the different groups of rats.

Time point	Normal control	Positive control	ASPM	Plain ASPM liposomes (LP-ASPM)	ASPM liposomes with PD-2 (LP-PD-2)
1 h	367 ± 38	712 ± 44*	121 ± 17*^$^	74 ± 10*^$#^	51 ± 9*^$#^
2 h	401 ± 39	656 ± 41*	118 ± 18*^$^	72 ± 8*^$#^	44 ± 6*^$#^
3 h	382 ± 35	679 ± 39*	110 ± 15*^$^	54 ± 6*^$#^	31 ± 5*^$#^

*p < 0.05 = significantly different from the normal control at the indicated time points.

$ *p* < 0.05 = significantly different from the positive control at the indicated time points.

# *p* < 0.05 = significantly different from ASPM at the indicated time points.

## Conclusion

In the present study, we developed ASPM liposomes conjugated with RGD and PDs for intestinal lymphatic targeting with the intention of enhancing the oral bioavailability of ASPM. We used custom-built PDs (lipidated and nonlipidated) for this study, and they were successfully synthesized, purified and characterized by SPPS. The DOEs of the ASPM-loaded liposomes were statistically optimized using the Box‒Behnken design, and the various parameters were characterized. The in vitro drug release profile of the liposomal formulation demonstrated the sustained release of ASPM. Liposomes showed appreciable viability in both Caco2 and Rajji-B cells; however, the cellular uptake of LP-PD-2 was greater than that of the other liposomes. The conjugated liposomes were primarily taken up by the cells via caveolae-based and energy-dependent endocytosis. Receptor saturation studies revealed a significant reduction in the uptake of conjugated liposomes after the receptor was saturated with RGD and PDs. LP-PD-2 showed superior PK parameters in rats, with an excellent reduction in L-DOPA-carbidopa-induced locomotor activity. In summary, ASPM liposome surface modified with custom-built novel PDs can target M cells in Peyer’s patches of the intestine and further enhance the bioavailability, PK and PD performance of ASPM.

## Electronic supplementary material

Below is the link to the electronic supplementary material.

Supplementary Material 1

## Data Availability

Data is presented in the main manuscript and Supplementary information.
