# Supplementary material for: Exploring peptide dendrimers for intestinal lymphatic targeting: formulation and evaluation of peptide dendrimer conjugated liposomes for enhancing the oral bioavailability of Asenapine maleate
Source: Sci Rep. 2024 Nov 15;14:28225. doi: 10.1038/s41598-024-79372-5 (PMC11568265; doi:10.1038/s41598-024-79372-5)
Supplement: Supplementary file 1 — Supplementary Material 1 [file 41598_2024_79372_MOESM1_ESM.docx]

**Supplementary Information**

**Exploring peptide dendrimers for intestinal lymphatic targeting: Formulation and evaluation of peptide dendrimer conjugated liposomes for enhancing the oral bioavailability of Asenapine maleate**

**Ajjappla Basavaraj Shreya^1^, Abhijeet Pandey^2^, Sanjay Kulkarni^1^, K Vijaya Bhaskar^3^, Harendra S Parekh^4^, Srinivas Mutalik^1*^**

^1^ Department of Pharmaceutics, Manipal College of Pharmaceutical Sciences, Manipal Academy of Higher Education, Manipal 576104, Karnataka, India

^2^ Formulation Research and Development, Global Drug Development/Technical Research and Development, Novartis Healthcare Pvt. Ltd., Genome Valley, Hyderabad 500101, Telangana, India

^3^ Department of Pharmaceutical Chemistry, Manipal College of Pharmaceutical Sciences, Manipal Academy of Higher Education, Manipal 576104, Karnataka, India

^4^ School of Pharmacy, Pharmacy Australia Centre of Excellence, The University of Queensland, Brisbane, QLD 4072, Australia

***Corresponding Author**

Dr Srinivas Mutalik

Principal and Professor

Manipal College of Pharmaceutical Sciences

Manipal Academy of Higher Education

Manipal 576104, Karnataka State, India

Email: [ss.mutalik@manipal.edu](mailto:ss.mutalik@manipal.edu)

## S1. Characterization of Peptide dendrimer

**
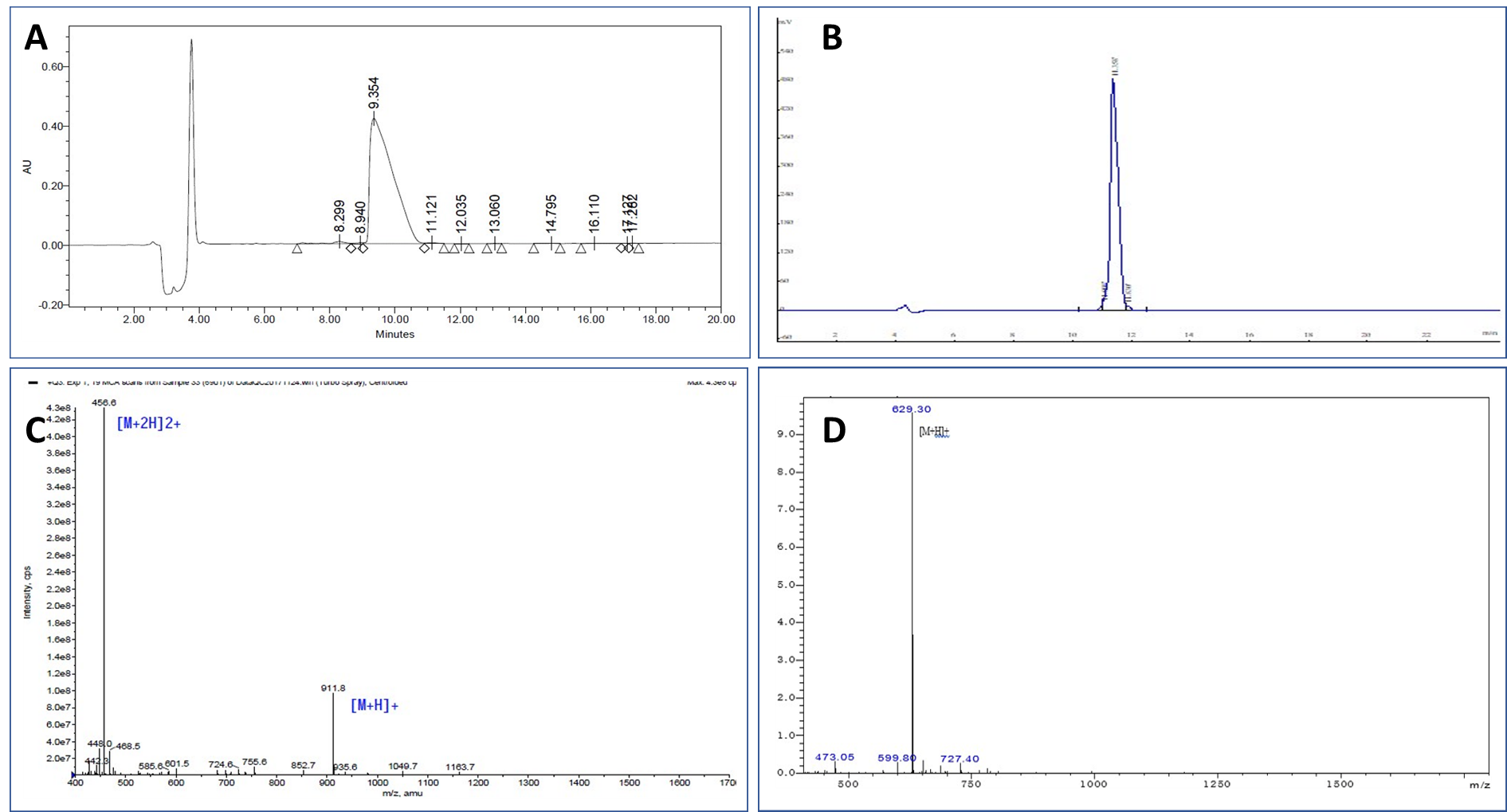
**

**Figure S1. HPLC chromatograms of (A) a lipidated peptide dendrimer (PD-1) and (B) a nonlipidated peptide dendrimer (PD-2). Mass spectra of the (C) lipidated peptide dendrimer (PD-1) and (D) nonlipidated peptide dendrimer (PD-2)**

**
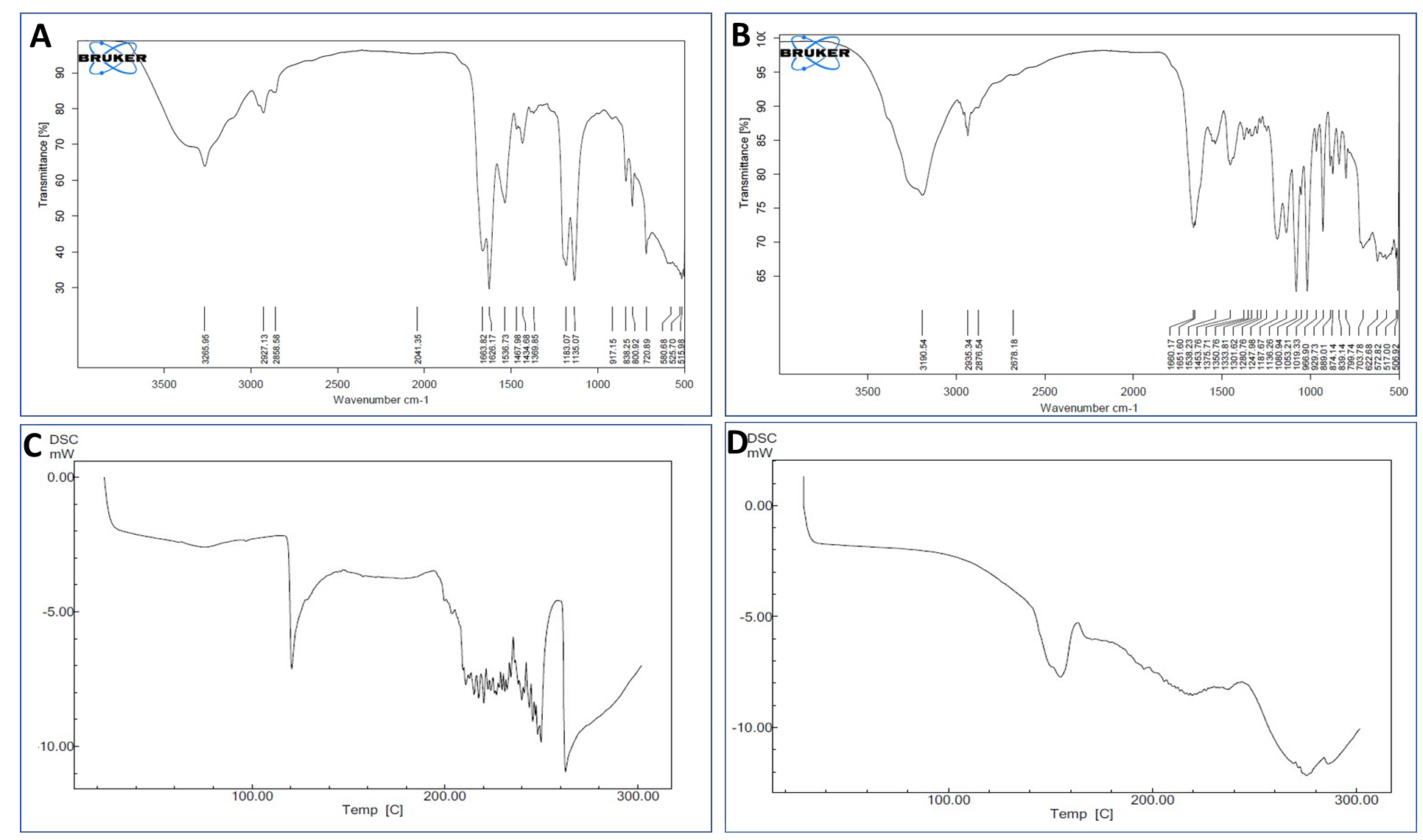
**

**Figure S2. FTIR spectra of (A) the lipidated peptide dendrimer (PD-1) and (B) the nonlipidated peptide dendrimer (PD-2). DSC thermograms of the (C) lipidated peptide dendrimer (PD-1) and (D) nonlipidated peptide dendrimer (PD-2)**

**
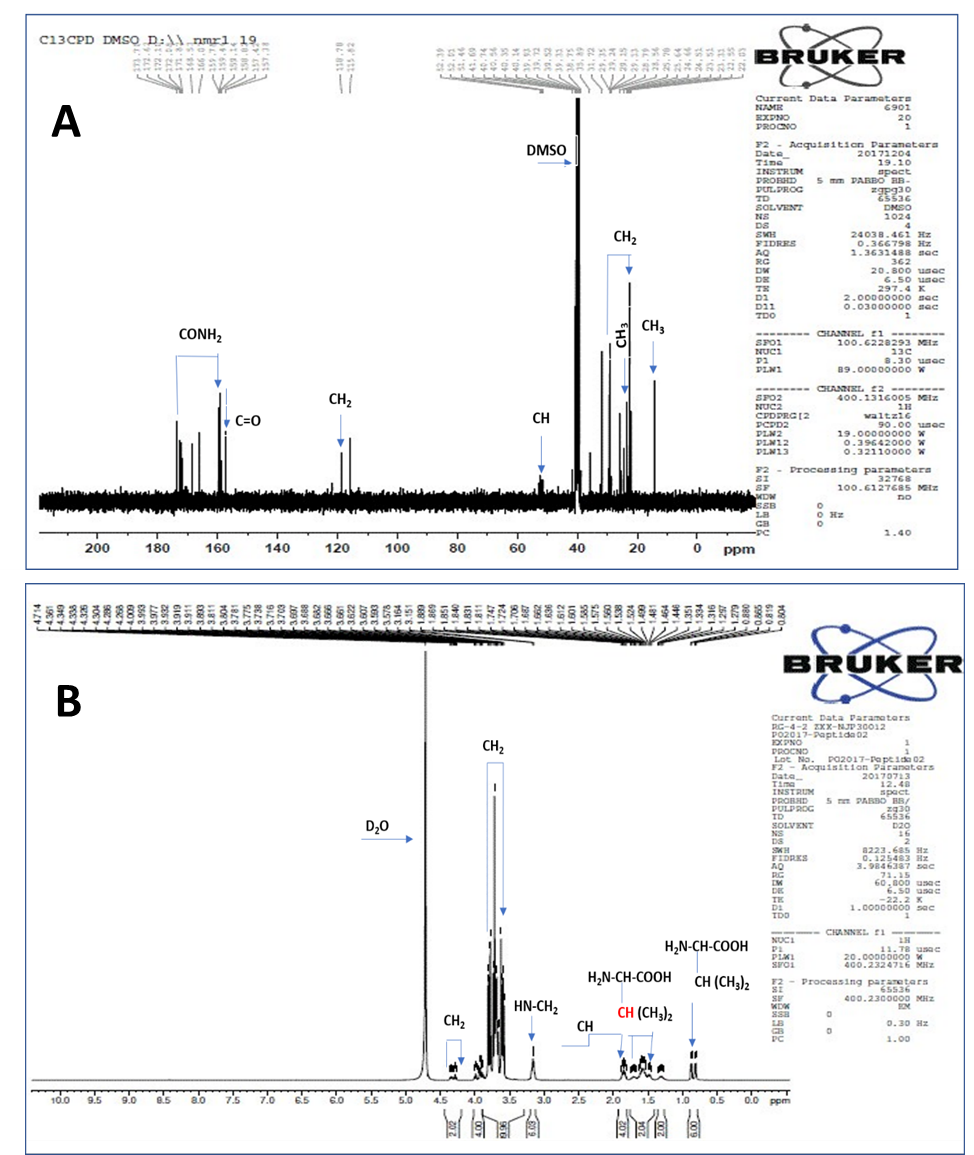
**

**Figure S3. NMR spectrum of the dendrimers. (A) ^13^C-NMR spectrum of a lipidated peptide dendrimer (PD-1). (B) ^1^H-NMR spectrum of a nonlipidated peptide dendrimer (PD-2).**

## S2. Analytical method development for the quantification of ASPM

An analytical HPLC method was developed and validated as per ICH Q2 (R1) guidelines to estimate the amount of ASPM present in the liposomal formulation. A reverse phase HPLC method was developed using the Shimadzu Prominance HPLC-20AT system (Shimadzu Corporation, Kyoto, Japan) liquid chromatography system configured with LC 20AD binary pumps, UV-visible detector, DGU-20A5 degasser unit, CTO-10AS column oven and SIL 20ACHT auto-sampler unit. LC solution software (LabSolution v.5.57) was used to monitor and process the chromatograms. The optimized chromatographic condition for the estimation of ASPM is given in Table S1.

**Table S1.** **Optimized chromatographic condition for the estimation of ASPM**

| **Chromatographic Conditions** | **Optimized parameters** |
| --- | --- |
| Method | Isocratic method |
| Column | Hyperclone BDS C18, 250 x 4.6 mm, 5µm particle size, 100Å pore size. |
| Mobile Phase A | Methanol – 5% |
| Mobile Phase B | Potassium phosphate buffer +0.1% v/v TEA (pH- 3±0.05)- 95% |
| Flow Rate | 0.8 mL/min |
| Column Temperature | 25 °C |
| Run time | 20 min |
| Diluent | Mobile phase A: B (5:95) |
| Detection wavelength | 230 nm |
| Injection Volume | 20 µL |
| Autosampler Temperature | 5 °C |


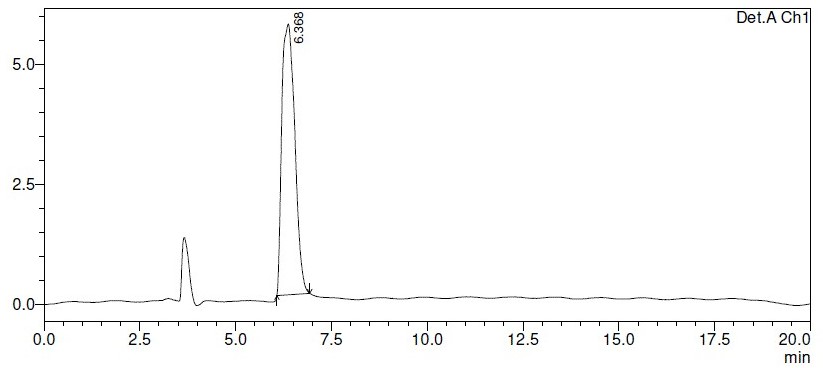


**Figure S4. Representative HPLC chromatogram of ASPM (t_R_- 6.3min)**

### HPLC analytical method validation: The developed analytical method was validated for parameters like linearity, system suitability, precision, accuracy, LOD and LOQ per ICH guidelines. The results of parameters validated is mentioned in Table S3.

1. ***System suitability:*** The system suitability parameters (Table S2) were calculated by injecting 1.5 µg/mL solution of ASPM six times into HPLC system.

**Table S2. System suitability parameters for the developed HPLC method of ASPM**

| **System suitability parameters** | **Acceptance criteria** | **Observed value** |
| --- | --- | --- |
| RSD of Peak area (n= 6) | RSD < 2.0% | 1.26 |
| Theoretical plate count | > 2000 | 19621 |

1. ***Specificity:*** To assess any interference with the blank at the retention time of ASPM, three replicates of the diluent as blank was injected into the chromatographic system.


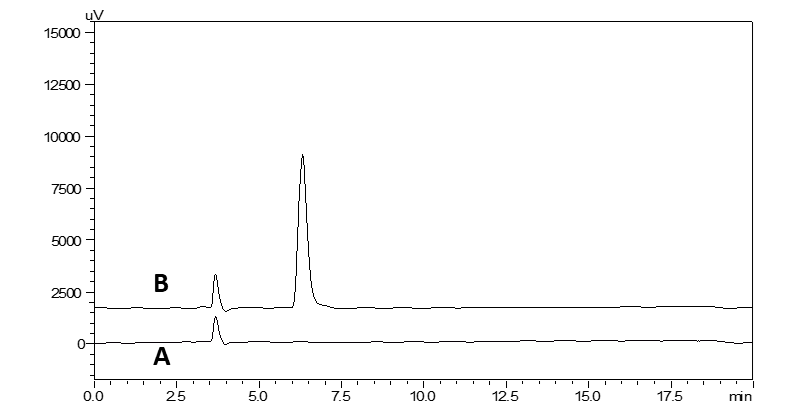


**Figure S5. Overlay chromatogram of A) blank diluent and B) ASPM standard solution**

**(10 µg/mL)**

1. ***Linearity:***  A single injection of each standard solutions of ASPM of concentration 0.2 – 20 µg/mL was injected into the HPLC system thrice using the optimized analytical method (Figure S6). The coefficient of determination (R^2^) was determined by plotting the calibration curve of peak area against corresponding concentration (µg/mL).
2. ***Determination of limit of detection (LOD) and limit of quantification (LOQ):*** LOD and LOQ were calculated as per ICH guidelines using the slope of the calibration curve and the standard deviation of ‘y’ intercept of regression lines.
3. ***Precision and Accuracy:*** The intra-day (Repeatability) and inter-day (Intermediate precision) precision were carried out on six replicate injections of 0.6 µg/mL, 10 µg/mL and 16 µg/mL of ASPM standard solution and %RSD was calculated. Accuracy was determined by calculating the percentage (%) recovery of ASPM at three different concentrations i.e., 7.5 µg/mL, 10 µg/mL and 12.5 µg/mL injected three times into the HPLC system.

**
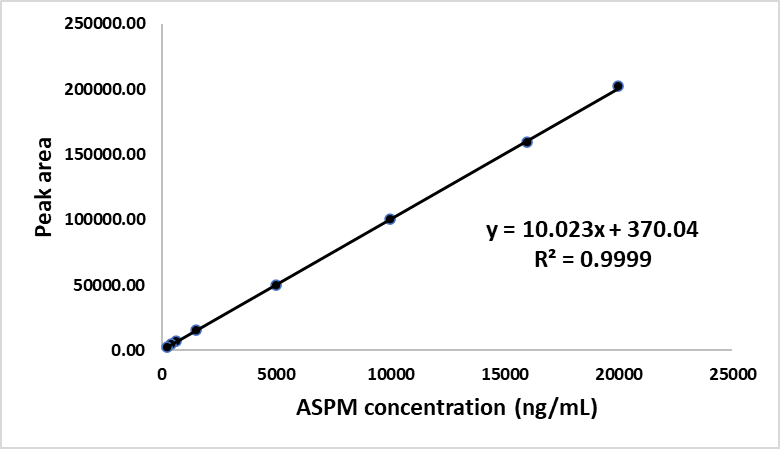
**

**Figure S6. Calibration curve of ASPM by RP-HPLC**

**Table S3. Results of analytical method validation parameters**

| **System suitability parameters** | System suitability parameters | Acceptance criteria | Observed | |
| --- | --- | --- | --- | --- |
|  | RSD of peak area (n=6) | RSD < 2.0% | 1.26 | |
|  | Theoretical plate count | > 2000 | 19621 | |
| **Linear regression data** | Linearity (µg/mL) (n=3) | 0.2 - 20µg/mL | | |
|  | Slope | 10.023 ± 2.22 | | |
|  | Y-Intercept when X=0 | 370.04± 351.68 | | |
|  | R^2^ | 0.9999 | | |
| **Precision** | % RSD for Intra-day | 0.23 (Acceptance criteria: <1.0) | | |
|  | % RSD for Inter-day | 0.41 (Acceptance criteria: <2.0) | | |
| **Accuracy** | Initial conc. (µg/mL) | Observed mean conc. (µg/mL) (n=3) | | Mean recovery (%) |
|  | 7.5 | 7.20 | | 96.11 |
|  | 10 | 10.25 | | 102.55 |
|  | 12.5 | 12.05 | | 96.43 |

**S3. Bioanalytical method development for the quantification of ASPM**

Reverse-phase HPLC method was developed for estimating the amount of ASPM present in rat plasma. The developed method was employed to study the drug's pharmacokinetics from the developed formulations. The HPLC conditions used for the analytical estimation methodology were tweaked off slightly for the analysis of ASPM in rat plasma. The optimised chromatographic conditions are given in Table S4 and the representative chromatograms in Figure S7.

**Extraction of ASPM from rat plasma and Preparation of blank plasma sample****:** The protein precipitation method was used to extract the drug from the rat plasma. Chilled acetonitrile was used as the protein precipitating agent. ASPM standard solution was prepared by dissolving 2 mg of ASPM in 2 mL of methanol and vortexed for 5 min to obtain a concentration of 1000 µg/mL. From this primary stock solution, the standard solution having a final concentration of 10 µg/mL was prepared by appropriately diluting the primary stock solution with the diluent. To prepare the standard solution of fexofenadine hydrochloride (FEXO) which was used as internal standard (IS), 2 mg of FEXO was dissolved in 50 µL of HPLC grade methanol and the volume was made up to 2 mL with Milli-Q water. From this stock solution, the standard solution having a final concentration of 10 µg/mL was prepared by appropriately diluting the primary stock solution with the diluent.

For linearity in plasma, calibration standards of ASPM in plasma were obtained by spiking the blank plasma with the working stock solutions (10 µL) of ASPM and FEXO working solution (10 µL) to get 50 to 2000 ng/mL concentrations of ASPM. A single injection of each calibration standard solutions of ASPM in plasma with a concentration ranging from 50 ng/mL to 2000 ng/mL was injected into the HPLC system thrice using the optimized bioanalytical method. The coefficient of determination (R^2^) was determined by plotting the peak area ratios of ASPM/ FEXO against the concentration of ASPM.

**Table S4. Results of Optimized chromatographic conditions for estimation of ASPM in rat plasma**

| **Chromatographic Conditions** | **Optimized parameters** |
| --- | --- |
| Method | Isocratic method |
| Column | Hyperclone BDS C18, 250 x 4.6 mm, 5µm particle size, 100Å pore size. |
| Mobile Phase A | Acetonitrile – 40% |
| Mobile Phase B | Ammonium acetate pH 4.5±0.05 - 60% |
| Flow Rate | 1.0 mL/min |
| Column Temperature | 25 °C |
| Run time | 15 min |
| Diluent | Mobile phase A: B (40:60) |
| Detection wavelength | 230 nm |
| Injection Volume | 80 µL |
| Autosampler Temperature | 5 °C |


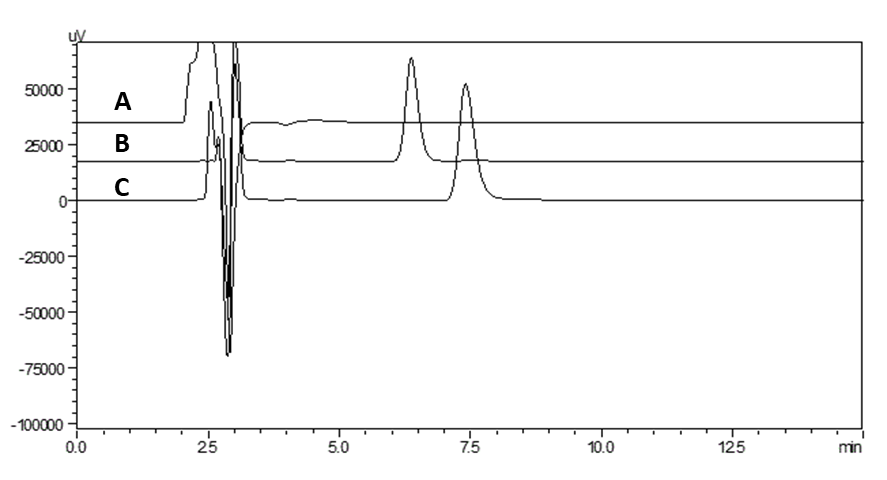


**Figure S7. Representative HPLC chromatograms of A) Blank Plasma, B) Fexofenadine hydrochloride (IS) and C) ASPM**

**S4. Drug- Excipient compatibility studies by FTIR.**

The compatibility between the ASPM and the excipients used in the formulation was studied using FT-IR. The samples were prepared by potassium bromide pellet method. 5 mg of the sample (ASPM, SPC, CHO, DPPC, PD-1, PD-2 alone separately and the physical mixture of ASPM+SPC+CHO+DPPC) were mixed with 200 mg of KBr and was compressed under a hydraulic press at 10,000 psi to form a transparent disc of 13 mm^2^ diameter. The spectra were obtained in the region of 4000 to 400 cm^-1^ by keeping the pellet in the light path.

The FTIR spectrum of cholesterol (Figure. S-8B) showed a characteristic intense peak at 2899 cm^-1^ which is due to CH_2_ symmetric stretching vibrations. The peaks present between 900 cm^-1^ to 675 cm^-1^ are mainly attributed to the C-H out of plane bending vibrations which are the characteristic pattern of aromatic substitution and the peak at 926 cm^-1^ is mainly due to the vibrational bending of =C-H group^1^. The FTIR spectrum of SPC (Figure. S-8C) showed characteristic absorption peaks at wavenumber 2922.65 cm^-1^, 2854.94 cm^-1^ and at 1735.98 cm^-1^ corresponding to the C-H and C=O vibrational stretching of the two long fatty acid tails present in SPC. The FTIR spectrum of DPPC (Figure. S-8D) showed a sharp intense peak at wavenumber 2849.73 cm^-1^, 1469.90 cm^-1^ and 1224.66 cm^-1^ corresponding to the CH_2_ stretching vibrations. At wavenumber 1731.87 cm^-1^, a sharp peak was observed corresponding to the carbonyl (C=O) group of DPPC. The sharp characteristic peak observed at 1093.32 cm^-1^ corresponds to PO_2_ stretching vibrations and the peak observed at wavenumber 969.15 cm^-1^ corresponds to N^+^-(CH_3_)_3_ groups of DPPC ^2^. The FTIR spectrum of physical mixture of ASPM, CHOL, DPPC and SPC (Figure. S-8E) showed the characteristic peaks of all the four components. The characteristic peaks of ASPM (Figure. S-8A) at wavenumbers 3011.31 cm^-1^ (C-H, SP^2^ - stretch), 1613.83 cm^-1^ (C=C), 1089.26 cm^-1^ (C-O), 860.21 cm^-1^ (C-H), 755.72 cm^-1^, 636.41 cm^-1^ and 579.08 cm^-1^ (C=O) were observed in the FTIR spectrum of the physical mixture. The presence of peaks in the range of 2820 cm^-1^ to 2980 cm ^-1^ representing the C-H stretching vibrations of lipids confirms the presence of lipids in the physical mixture ^3^. The presence of the majority of the peaks of lipids and ASPM in the FTIR spectrum of physical mixture indicates compatibility and lack of any chemical interactions between the drug and excipients used in the formulation.


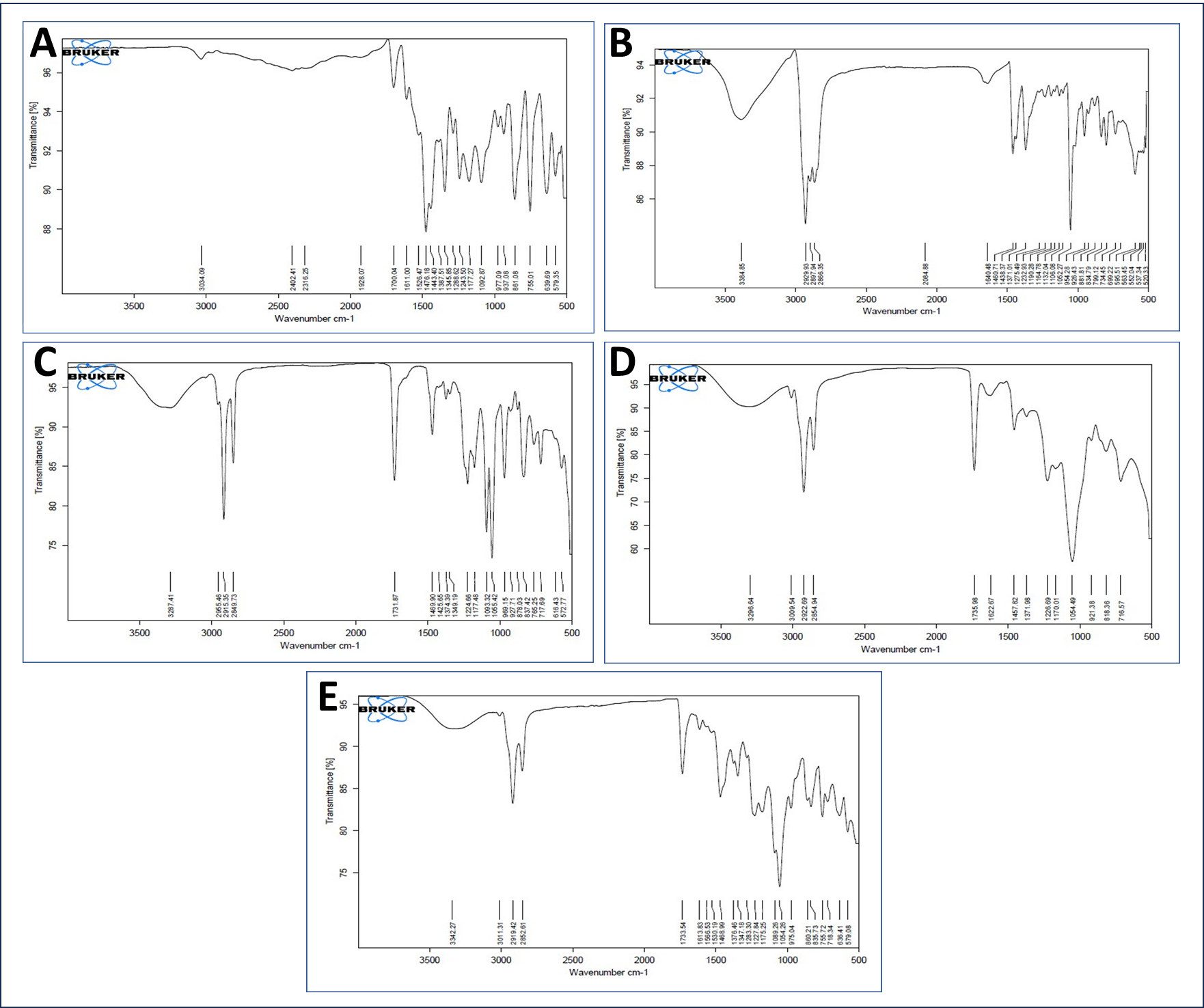


Figure. S8. FTIR spectra of different samples

A) ASPM, B) Cholesterol, C) SPC, D) DPPC and E) Physical mixture of ASPM and excipients

**S5. Screening of process variables by one factor at a time (OFAT) method**

The effect of process variables *viz*. types of phospholipids, composition of phospholipid, total lipid content, hydration media, rotary flash evaporator parameters, probe sonication time and probe sonication pulser “on time” on particle size, PDI and ZP were studies by employing OFAT method.

**S5a. Effect of types of phospholipids**

The results of effect of type of phospholipid on liposome parameters is given in Table S5. It is evident that, the liposome prepared with DPPC as an additional phospholipid exhibited a lower particle size than the liposomes prepared with SPC+CHOL. There was not much difference in the values of ZP and PDI among two different lipid compositions. Thus, depending on the particle size, SPC+CHOL+DPPC was selected as the combination of phospholipids for the formulation of liposomes. The use of DPPC in the preparation of liposomes is known to reduce the particle size as DPPC will probably induce the reorganization of the liposomal bilayer, thus enhancing the surface area and thereby reducing the particle size. Furthermore, DPPC undergoes phase transition at 41℃ and promotes a higher drug release and permeation from the liposomal drug delivery system ^4,5^. Using cholesterol along with SPC and DPPC provides additional rigidity to the lipid bilayer as cholesterol fills into the empty spaces between the phospholipid molecules and anchors them more strongly into the liposomal membrane, thereby enhancing the stability of the bilayer.

**Table S5. Results of effect of type of phospholipid on liposome parameters**

| **Type of Phospholipid** | **Particle size (nm)** | **ZP (mv)** | **PDI** |
| --- | --- | --- | --- |
| SPC + CHOL | 143.2 ± 1.9 | -47.6 ± 2.5 | 0.134 ± 0.0 |
| SPC + CHOL + DPPC | 102.8 ± 1.9 | -45.07 ± 3.00 | 0.24 ± 0.06 |

The results are presented as Mean ± SD, n=3

**S5b. Effects of phospholipid ratio**

It was observed that DPPC and CHOL ratio had a profound effect on the particle size of the liposomes (Table S6). An increase in the particle size was observed in the batches where the concentration of DPPC and cholesterol was kept same, whereas a considerable decrease in the particle size of liposomes was observed in the batch where DPPC concentration was increased compared to the concentration of cholesterol. However, the phospholipid ratio did not have much effect on the ZP and PDI of the liposomes. The reduction in the particle size with the increase in DPPC concentration could be because of the fact that DPPC promotes rigidity of the bilayer and enhances colloidal stability, thus decreasing the particle size

**Table S6. Results of effect of phospholipid ratio on liposomal parameters**

| **Ratio of Phospholipids (SPC: DPPC: CHOL)** | **Particle size (nm)** | **ZP (mv)** | **PDI** |
| --- | --- | --- | --- |
| 1: 0.125: 0.125 | 154.7 ± 1.15 | -40.7 ± 0.56 | 0.253 ± 0.03 |
| 1: 0.25: 0.125 | 105.7 ± 4.31 | -44.2 ± 1.21 | 0.21 ± 0.01 |
| 1: 0.25: 0.25 | 121.4 ± 2.07 | -44.8 ± 3.14 | 0.21 ± 0.03 |

The results are presented as Mean ± SD, n=3

**S5c. Effect of total lipid content**

As the total lipid content increased, a decline in the particle size was observed, while, no definite trend was observed with respect to PDI (Table S7). However, a noteworthy reduction in the ZP was observed with LP-1, which may be due to the presence of a smaller number of phosphate groups at the surface of the liposome. The reduction in the particle size with the increase in lipid content (LP-4) may be because of the reorganization of the lipid bilayer, resulting in a greater number of narrow-sized small particles ^6^.

**Table S7. Results of the effect of total lipid content on liposome parameters**

| **Batches** | **Total lipid content (mg)** | **Particle size (nm)** | **PDI** | **ZP (mv)** |
| --- | --- | --- | --- | --- |
| LP-1 | 50 | 160.83 ± 1.46 | 0.36 ± 0.03 | -31.43 ± 1.19 |
| LP-2 | 100 | 175.37 ± 2.08 | 0.38 ± 0.01 | -54.37 ± 1.97 |
| LP-3 | 150 | 130.9 ± 1.91 | 0.27 ± 0.05 | -54.20 ± 1.77 |
| LP-4 | 200 | 111 ± 0.8 | 0.251 ± 0.04 | -50.47 ± 1.58 |

The results are presented as Mean ± SD, n=3

**S5d. Effect of sonication time**

An inversely proportional relationship between the sonication time and particle size was observed i.e., a decrease in the particle size was observed with the increase in the sonication time from 5 min to 9 min (Table S8). However further increase in the sonication time to 10 min, resulted in increased particle size of the liposomes. The PDI also showed the same trend as the particle size of liposomes, and there was no considerable effect on the ZP of the liposomes. The acoustic cavitation, which is the main phenomenon of sonication that happens all over the solution, induces very large shear forces on the particles, thereby breaking the particles into smaller ones. The increase in sonication time increases the cavitation effect on the particles for a prolonged period of time as the particles are exposed to the ultrasonic waves for an extensive time, thus reducing the size of the liposomes ^7^.

**Table S8. Results of the effect of sonication time on liposome parameters**

| **Batches** | **Sonication time (min)** | **Particle size (nm)** | **PDI** | **ZP (mV)** |
| --- | --- | --- | --- | --- |
| LP-A | 5 | 189.7 ± 1.31 | 0.43 ± 0.01 | -49.93 ± 0.64 |
| LP-B | 6 | 155.2 ± 1.25 | 0.33 ± 0.01 | -52.93 ± 2.45 |
| LP- C | 8 | 132.3 ± 0.95 | 0.138 ± 0.01 | -52.00 ± 2.02 |
| LP- D | 9 | 115.1 ± 2.44 | 0.238 ± 0.01 | -49.6 ± 2.44 |
| LP -E | 10 | 148.07 ± 5.01 | 0.21 ± 0.04 | -54.73 ± 0.57 |

The results are presented as Mean ± SD, n=3

**S5e. Effect of pulser “on time”**

It is evident that as the pulse rate increased from 2 sec to 8 sec, a decrease in the particle size was observed, and there was no significant effect of the pulse rate on PDI and ZP (Table S9). The reduction in the particle size can be attributed to the fact that the cavitation activity of the liposomal suspension can be enhanced by adequately controlling the pulse on and off time, resulting in maximizing the active cavitation of the bubble population of the sample. Furthermore, the fluid flow created by the ultrasound waves will have more time to relax in the pulsated irradiation mode, resulting in lower flow rates and decreasing particle size ^8,9^.

**Table S9. Results of effect of pulser “on time” on liposome parameters**

| **Batches** | **Pulser “on time” (sec)** | **Particle size (nm)** | **PDI** | **ZP (mv)** |
| --- | --- | --- | --- | --- |
| LP- F | 2 | 131.6 ± 1.89 | 0.15 ± 0.01 | -47.20 ± 1.60 |
| LP- G | 4 | 123.3 ± 2.9 | 0.23 ± 0.04 | -53.77 ± 3.27 |
| LP- H | 6 | 121.7 ± 1.80 | 0.32 ± 0.02 | -54.13 ± 3.31 |
| LP- I | 8 | 112.4 ± 1.88 | 0.28 ± 0.01 | -56.2 ± 2.23 |

The results are presented as Mean ± SD, n=3

**S5f. Effect of hydration media**

Liposome batches with distilled water and pH 7.4 phosphate buffer (PB) as hydration media were prepared. It was observed that the hydration media did not exert any substantial effect on the liposome parameters like particle size, ZP and PDI (Table S10). However, pH 7.4 PB was selected as the hydration medium as it aids in the particle formation, provides suitable ion concentration and osmolarity that prevents the rupturing of the vesicles and also stabilizes the drug present in the vesicles ^10^.

**Table S10. Selected process variables for the formulation of ASPM liposomes**

| **Sl. No.** | Factors | **Optimized value** |
| --- | --- | --- |
| 1 | Type of phospholipid | SPC: DPPC: CHOL |
| 2 | Composition of phospholipids | 1:0.25:0.125 |
| 3 | Total lipid content | 200 mg |
| 4 | Probe sonication time | 9 min |
| 5 | Probe sonicator pulser “on time” | 8 sec |
| 6 | Hydration media | pH 7.4 PB |
| 7 | Rotary evaporator parameters | 45 ℃/ 80 rpm |

S6. Optimisation of ASPM-loaded liposomes

**S6a. Experimental design**

**Table S11. Independent variables and their coded levels in the Box–Behnken design**

| **Factors** | **Levels** | | |
| --- | --- | --- | --- |
|  | **Low (-1)** | **Middle (0)** | **High (+1)** |
| Total lipid content (mg) | 100 | 150 | 200 |
| Sonication time (min) | 5 | 9 | 13 |
| Pulser on (sec) | 6 | 8 | 10 |

**Table S12. Experimental batches of the liposomal formulation in the Box–Behnken design**

| **Batches** | **Total lipid**  **Content (mg)** | **Sonication time (min)** | **Pulser on (sec)** |
| --- | --- | --- | --- |
| LP-ASPM-F1 | 150 | 5 | 10 |
| LP-ASPM-F2 | 200 | 9 | 10 |
| LP-ASPM-F3 | 150 | 9 | 8 |
| LP-ASPM-F4 | 150 | 5 | 6 |
| LP-ASPM-F5 | 100 | 9 | 6 |
| LP-ASPM-F6 | 150 | 13 | 10 |
| LP-ASPM-F7 | 100 | 13 | 8 |
| LP-ASPM-F8 | 100 | 5 | 8 |
| LP-ASPM-F9 | 100 | 9 | 10 |
| LP-ASPM-F10 | 150 | 13 | 6 |
| LP-ASPM-F11 | 200 | 9 | 6 |
| LP-ASPM-F12 | 150 | 9 | 8 |
| LP-ASPM-F13 | 200 | 13 | 8 |
| LP-ASPM-F14 | 200 | 5 | 8 |
| LP-ASPM-F15 | 150 | 9 | 8 |

**S6b. Effect of independent factors on particle size**

**Table S13. ANOVA results for the effects of independent factors on particle size**

**
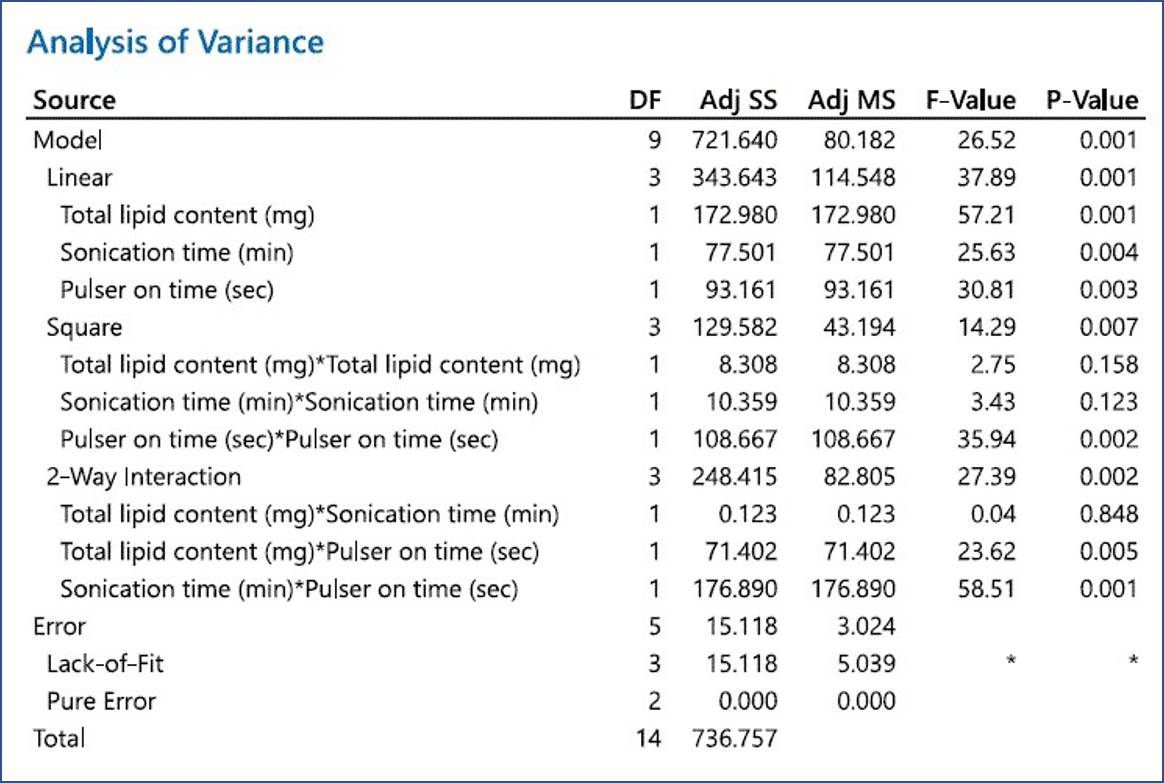
**

**
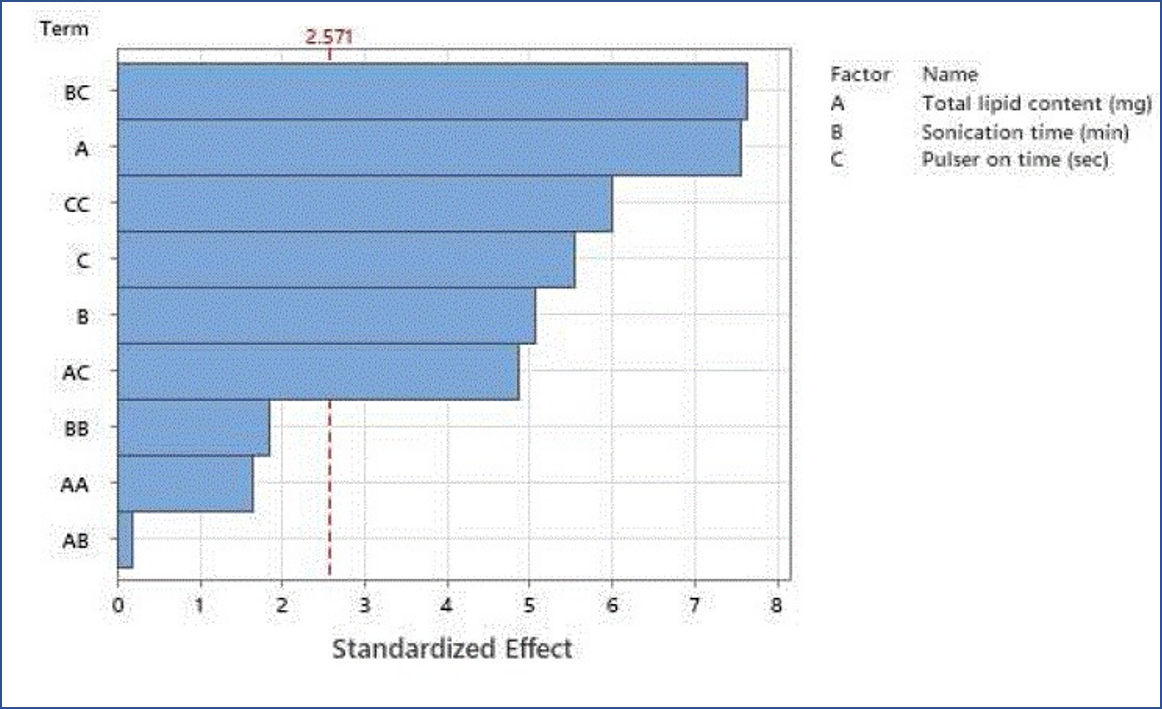
**

# Figure S9. Pareto chart for the effect of independent factors on particle size

**
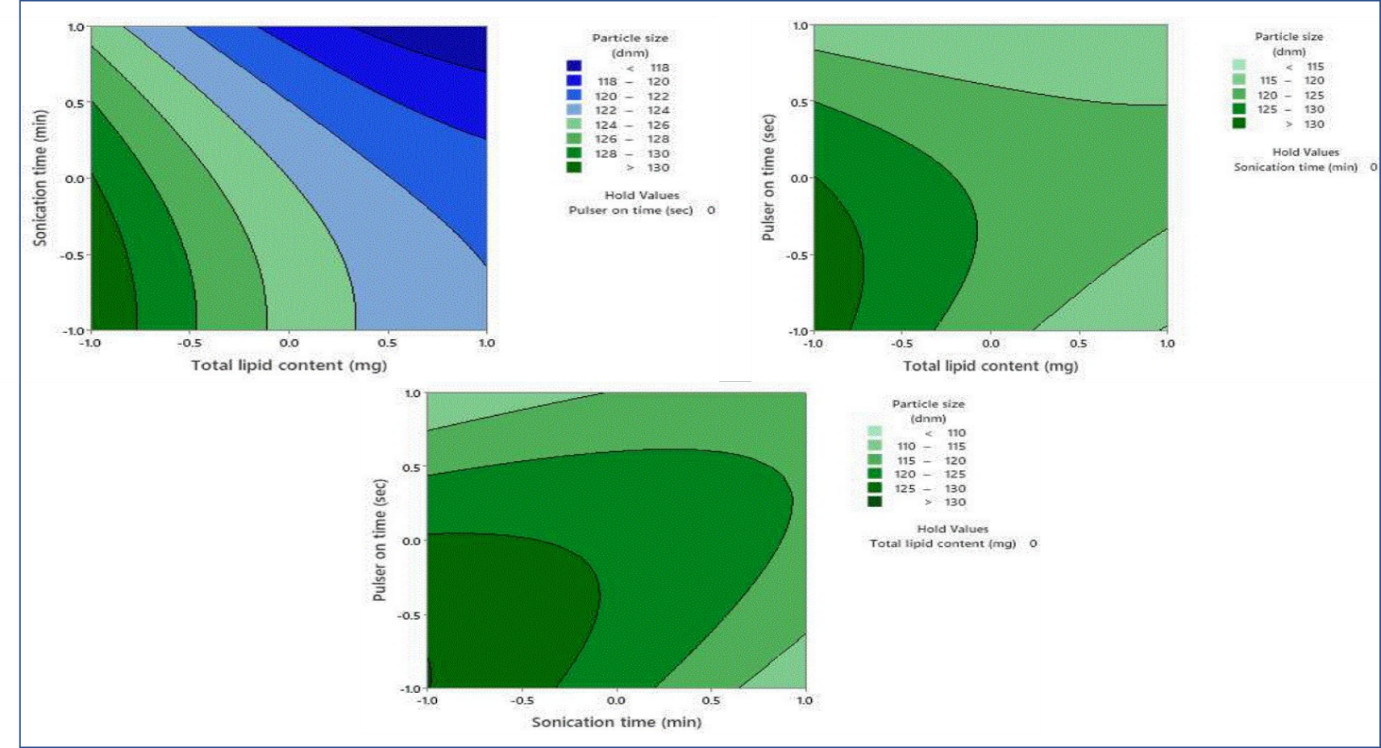
Figure S10. Contour plots of the particle size *vs* the independent variable.**

**B**

**C**

**A: Particle size *vs* total lipid content and sonication time, B: Particle size *vs* total lipid content and pulser “on time” and C: Particle size *vs* sonication time and pulser “on time”.**

**
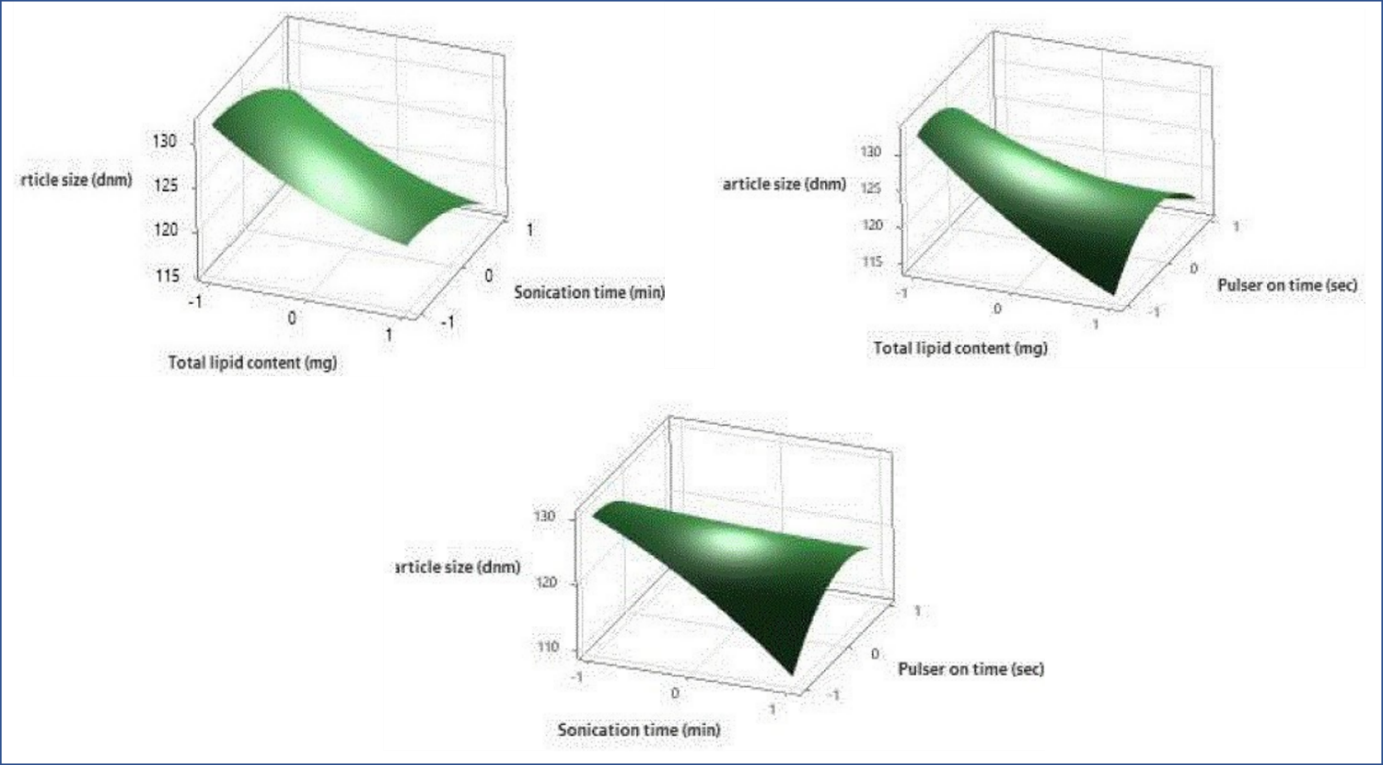
Figure S11. Surface plots of particle size *vs.* independent factors.**

**A: Particle size *vs* total lipid content and sonication time, B: Particle size *vs* total lipid content and pulser “on time” and C: Particle size *vs* sonication time and pulser “on time”.**

**S6c. Effect of independent factors on entrapment efficiency**

# Table S14. ANOVA results for the effects of independent factors on entrapment efficiency

**
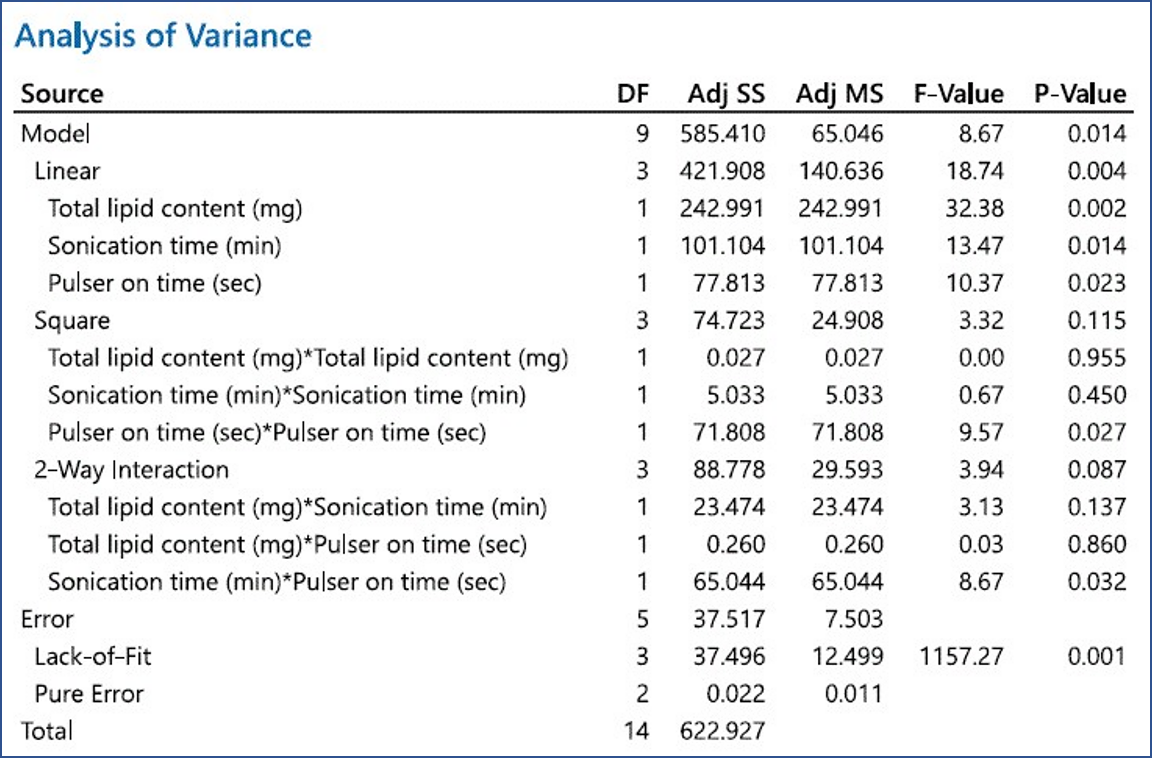
**

**
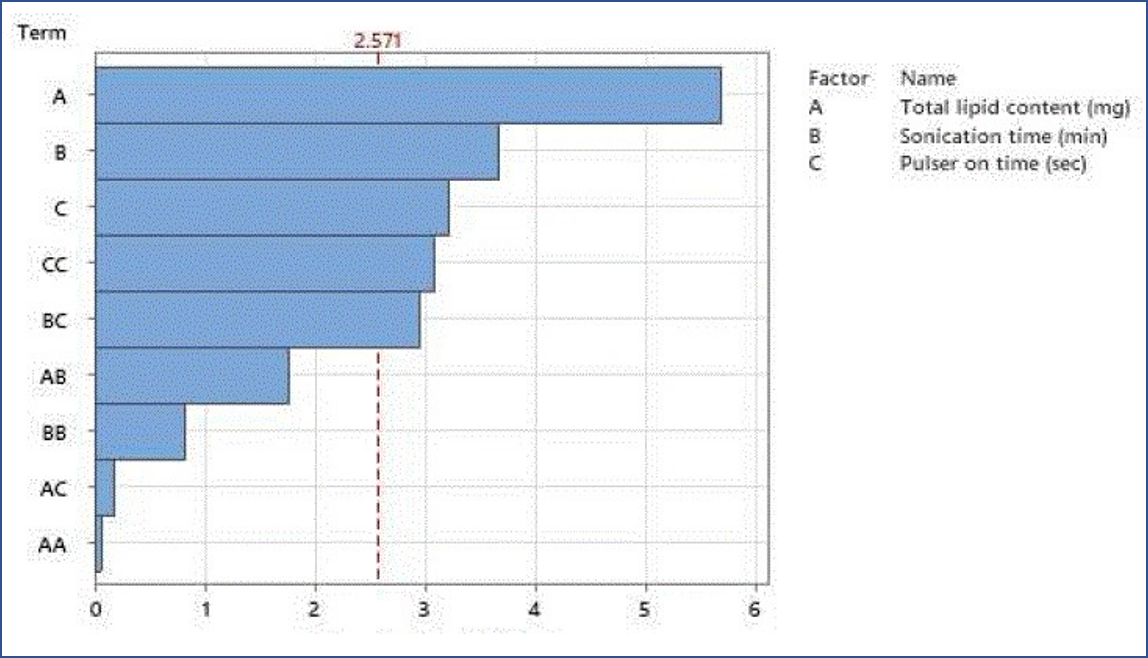
**

## Figure S12. Pareto chart for the effect of independent factors on entrapment efficiency

**
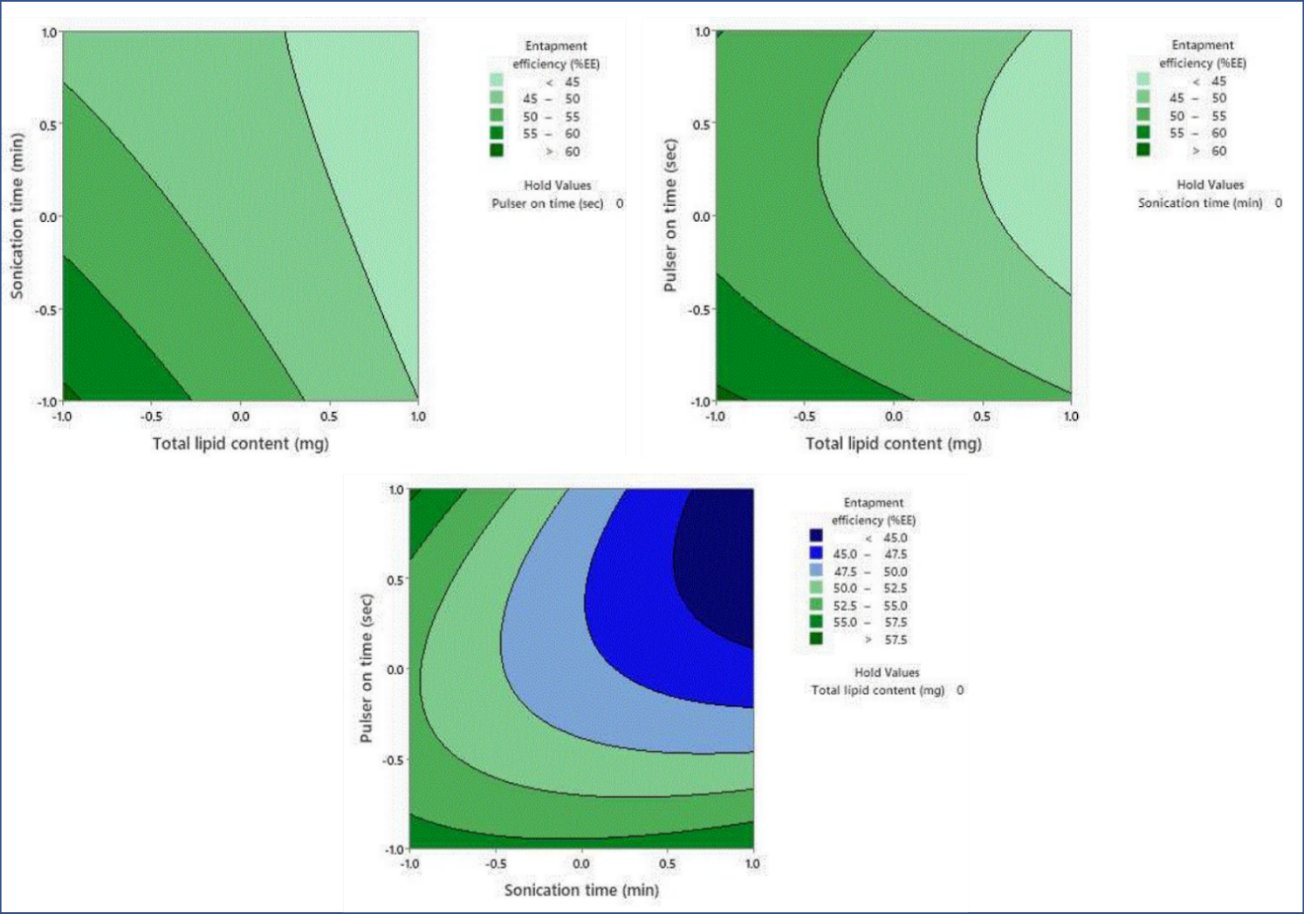
**

**C**

**B**

**A**

**Figure S13. Contour plots of entrapment efficiency *vs* the independent variable.**

**A: Entrapment efficiency *vs* total lipid content and sonication time, B: Entrapment efficiency *vs* total lipid content and pulser “on time” and C: Entrapment efficiency *vs* sonication time and pulser “on time”.**

**
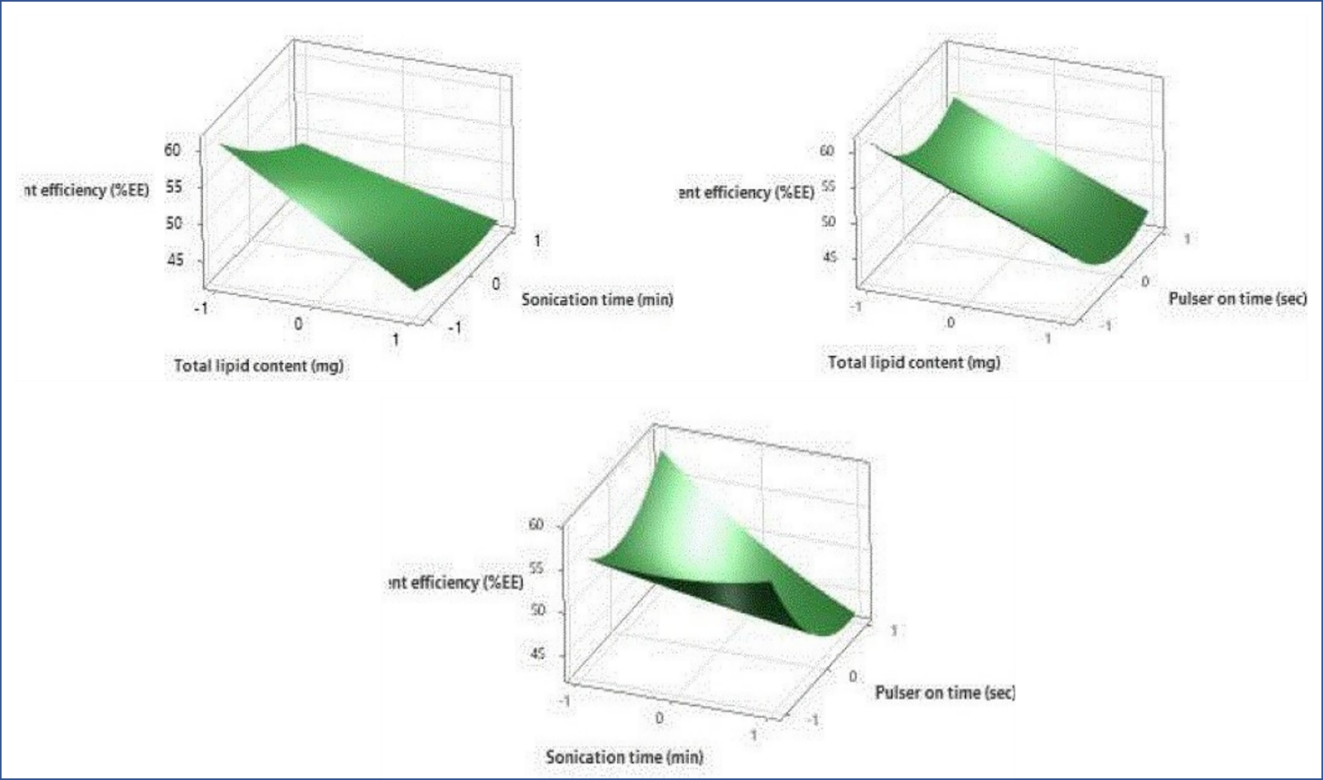
Figure S14. Surface plots of entrapment efficiency *vs* independent factors.**

**C**

**B**

**A**

**A: Entrapment efficiency *vs* total lipid content and sonication time, B: Entrapment efficiency *vs* total lipid content and pulser “on time and C: Entrapment efficiency *vs* sonication time and pulser “on time”.**

**
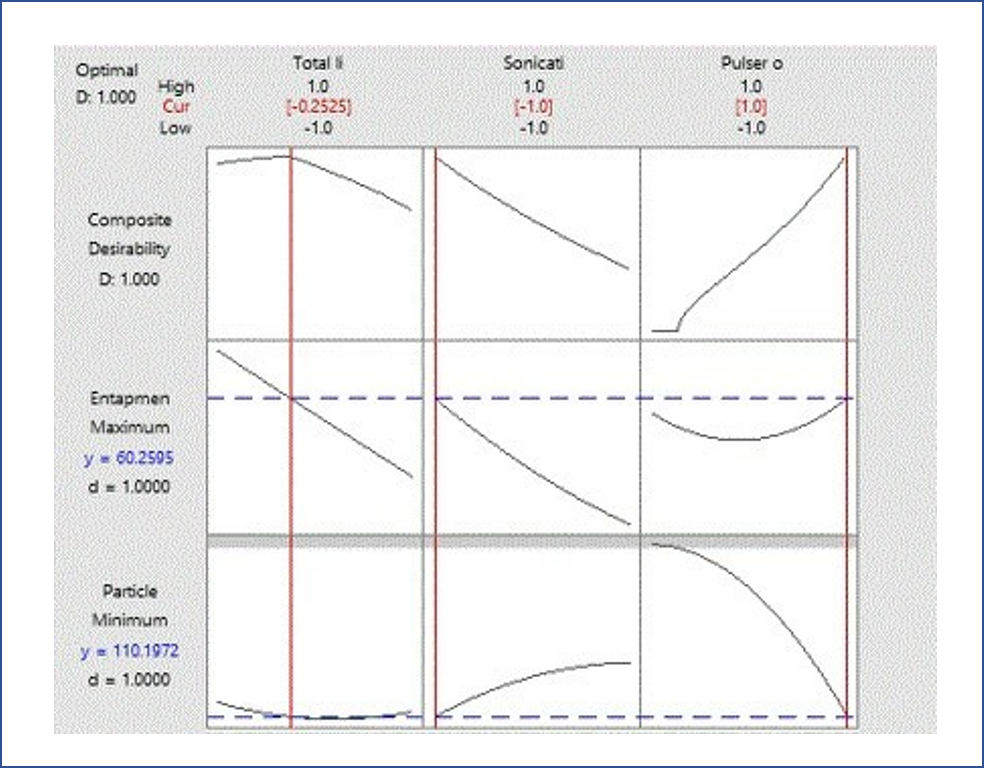
 Figure S15. Desirability plot in the Box–Behnken design**

**S6d. Optimised design validation**

Table S15. Predicted and observed values for responses with % relative error in the Box‒Behnken design

| **Response** | **Predicted mean ± SD** | **Predicted 95% PI** | **Observed mean ± SD (n=3)** | **% Relative error** |
| --- | --- | --- | --- | --- |
| Size (nm) | 110.20 ± 2.41 | 104.24 to 116.15 | 112.9 ± 1.1 | 2.4 |
| %EE | 60.26 ± 1.53 | 50.88 to 69.64 | 57.2 ± 1.6 | 5.07 |

**S7. Effect of amount of drug and different cryoprotectants on the optimized liposomes.**

**Table S16. Effect of amount of drug on liposome parameters**

| **Batches** | **Drug (mg)** | **Particle size (nm)** | **PDI** | **ZP (mv)** | **EE (%)** | **LE (%)** |
| --- | --- | --- | --- | --- | --- | --- |
| LP-OPT-10 | 10 | 113.23 ± 0.49 | 0.28 ± 0.02 | -51.59 ± 1.21 | 85.80 ± 1.41 | 4.20 ± 0.10 |
| LP-OPT-15 | 15 | 111.73 ± 1.10 | 0.27 ± 0.05 | -50.90 ± 0.71 | 84.13 ± 0.82 | 6.24 ± 0.09 |

**Table S17. Effect of different cryoprotectants on liposome parameters**

| **Batches** | **Particle size (nm)** | **PDI** | **ZP (mv)** |
| --- | --- | --- | --- |
| **Before Lyophilization** | | | |
| LP-OPT-T | 112.9 ± 1.85 | 0.24 ± 0.02 | - 49.21 ± 1.32 |
| LP -OPT- M | 112.9 ± 1.85 | 0.24 ± 0.02 | - 49.21 ± 1.32 |
| LP-OPT- S | 112.9 ± 1.85 | 0.24 ± 0.02 | - 49.21 ± 1.32 |
| **After Lyophilization** | | | |
| LP-OPT- T | 164.16 ± 2.00 | 0.32 ± 0.01 | - 52.01 ± 2.03 |
| LP- OPT- M | 540.6 ± 1.70 | 0.50 ± 0.10 | - 54.07 ± 1.70 |
| LP-OPT- S | 473.1 ± 2.04 | 0.527 ± 0.10 | - 56.23 ± 1.60 |

The results are presented as Mean±SD, n=3; T- Trehalose, S- Sucrose and M- Mannitol

**S8. Surface modification of LP-ASPM by EDC-NHS chemistry.**

**Table S18. Effect of DSPE-PEG-COOH_2000_ concentration on liposome parameters**

| **Batch** | **DSPE-PEG-COOH_2000_** | **Size (nm)** | **PDI** | **ZP (mv)** |
| --- | --- | --- | --- | --- |
| LP-DSPE-PEG-2% | 2% | 82.21 ± 1.38 | 0.28 ± 0.05 | -45.79 ± 0.54 |
| LP-DSPE-PEG-5% | 5% | 115.3 ± 1.22 | 0.33 ± 0.10 | -39.39 ± 0.74 |
| LP-DSPE-PEG-8% | 8% | 111.27 ± 1.17 | 0.39 ± 0.03 | -37.4 ± 1.29 |
| LP-DSPE-PEG-10% | 10% | 88.02 ± 1.05 | 0.30 ± 0.03 | -37.63 ± 1.36 |

Results are presented as Mean±SD, n=3

**S9. Characterization of unconjugated and conjugated ASPM liposomes**

**Table S19. Liposome parameters of conjugated and unconjugated liposomes.**

| **Batches** | **Particle size (nm)** | **PDI** | **ZP (mv)** | **EE (%)** |
| --- | --- | --- | --- | --- |
| LP-ASPM | 111.73 ± 1.10 | 0.27 ± 0.05 | -51.59 ± 1.21 | 84.13 ± 0.82 |
| LP-RGD | 122.2 ± 1.57 | 0.153 ± 0.01 | -52.19 ± 1.44 | 75.00 ± 4.06 |
| LP- PD-1 | 131.4 ± 1.04 | 0.242 ± 0.02 | 33.16 ± 1.57 | 81.81 ± 1.65 |
| LP- PD-2 | 125.43 ± 1.80 | 0.199 ± 0.05 | 32.12 ± 1.92 | 81.14 ± 1.38 |

LP-ASPM- Liposomes with ASPM, LP-RGD- ASPM liposomes conjugated with RGD, LP-PD-1- ASPM liposomes conjugated with PD-1 (Lipidated PD) and LP-PD-2- ASPM liposomes conjugated with PD-2 (Non- lipidated PD).

##### S10. DSC analysis of different formulations

**
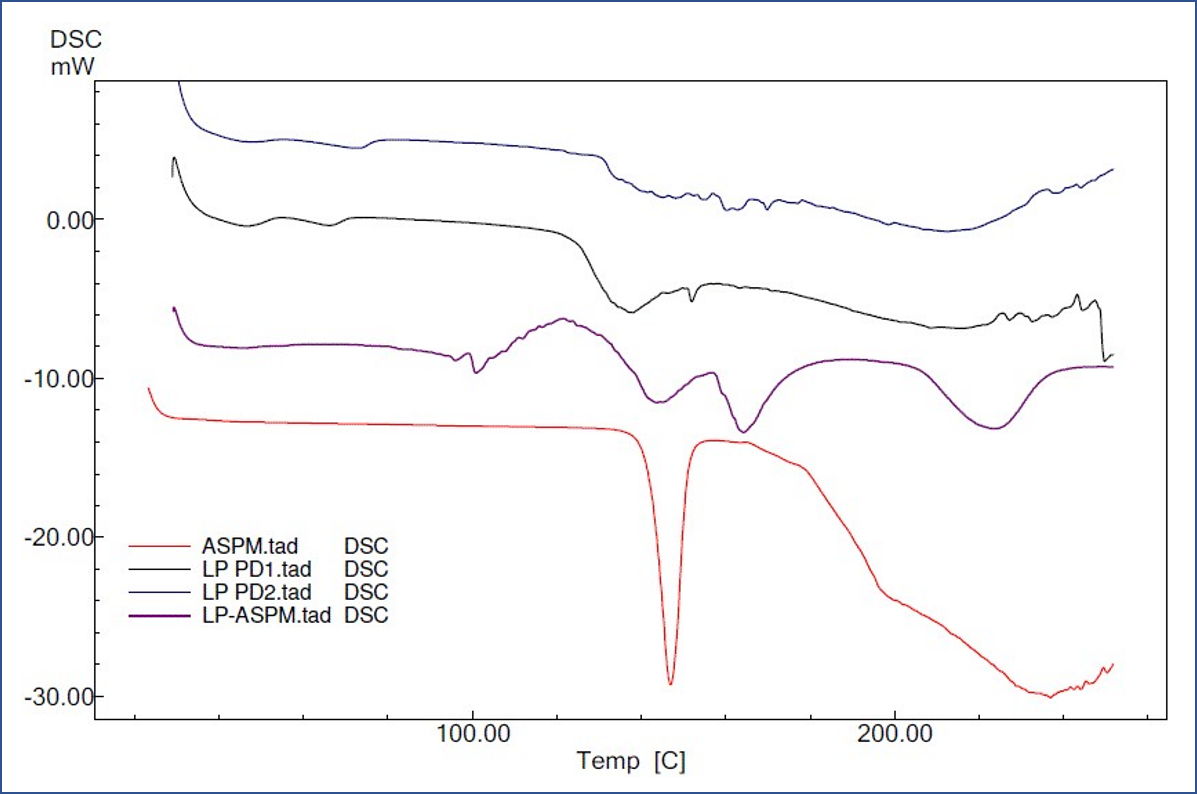
**

**D**

**C**

**B**

**A**

**Figure S16. DSC thermograms of A) ASPM, B) LP-ASPM, C) LP-PD-1 and D) LP-PD-2**

**S10. Pharmacokinetic studies**

**Table S20. The pharmacokinetic parameters of Plain ASPM, LP-ASPM and LP-PD-2 when administered orally in rats.**

| **Parameters** | **ASPM** | **LP-ASPM** | **LP-PD-2** |
| --- | --- | --- | --- |
| Cmax (ng/mL) | 130.66 ± 7.01 | 198.68 ± 1.237* | 297.89 ± 16.67*^#^ |
| Tmax (h) | 1.00± 0.00 | 4.00±0.00* | 4.00±0.00* |
| AU_C0-t_ (h*ng/mL) | 2658.63 ± 220.89 | 3602.53 ± 331.67* | 5081.24 ± 378.66*^#^ |
| AUC_0-∞_ (h*ng/mL) | 3636.67 ± 346.78 | 5916.29 ± 402.46* | 8855.68 ± 567.56*^#^ |
| t_1/2_ (h) | 25.52 ± 2.15 | 44.48 ± 3.26* | 55.91 ± 3.89*^#^ |
| K_el_ (1/h) | 0.0271 ± 0.002 | 0.016 ± 0.001* | 0.012 ± 0.001*^#^ |
| MRT (h) | 36.00 ± 3.16 | 54.18 ± 4.16* | 64.03 ± 4.78*^#^ |

All values are expressed as mean ± SD; n = 6

* Indicates statistically significant (*p*<0.05) when compared to ASPM.

# Indicates statistically significant (*p*<0.05) when compared to LP-ASPM.

Cmax: Maximum concentration; Tmax: Time for maximum concentration; AUC: Area under the curve, t_1/2_: Elimination half-life, Kel: Elimination rate constant, MRT: Mean residential time

**References**

1. Gupta, U., Singh, V. K., Kumar, V. & Khajuria, Y. Spectroscopic studies of cholesterol: fourier transform infra-red and vibrational frequency analysis. *Mater. Focus* **3**, 211–217 (2014).

2. Kotanen, C. N., Moussy, F. G., Carrara, S. & Guiseppi-Elie, A. Implantable enzyme amperometric biosensors. *Biosens. Bioelectron.* **35**, 14–26 (2012).

3. Simonova, D. & Karamancheva, I. Application of Fourier transform infrared spectroscopy for tumor diagnosis. *Biotechnol. Biotechnol. Equip.* **27**, 4200–4207 (2013).

4. Mufamadi, M. S. *et al.* A review on composite liposomal technologies for specialized drug delivery. *J. Drug Deliv.* **2011**, (2011).

5. Tefas, L. R. *et al.* Quercetin-loaded liposomes: formulation optimization through a D-optimal experimental design. *Farmacia* **63**, 26–31 (2015).

6. Soema, P. C., Willems, G.-J., Jiskoot, W., Amorij, J.-P. & Kersten, G. F. Predicting the influence of liposomal lipid composition on liposome size, zeta potential and liposome-induced dendritic cell maturation using a design of experiments approach. *Eur. J. Pharm. Biopharm.* **94**, 427–435 (2015).

7. Esmaeilzadeh-Gharedaghi, E. *et al.* Effects of processing parameters on particle size of ultrasound prepared chitosan nanoparticles: An Artificial Neural Networks Study. *Pharm. Dev. Technol.* **17**, 638–647 (2012).

8. Gielen, B. *et al.* Energy efficient crystallization of paracetamol using pulsed ultrasound. *Chem. Eng. Process. Process Intensif.* **114**, 55–66 (2017).

9. Rouxel, D., Hadji, R., Vincent, B. & Fort, Y. Effect of ultrasonication and dispersion stability on the cluster size of alumina nanoscale particles in aqueous solutions. *Ultrason. Sonochem.* **18**, 382–388 (2011).

10. Obeid, M. A., Khadra, I., Mullen, A. B., Tate, R. J. & Ferro, V. A. The effects of hydration media on the characteristics of non-ionic surfactant vesicles (NISV) prepared by microfluidics. *Int. J. Pharm.* **516**, 52–60 (2017).
